# Deciphering the role of zinc homeostasis in the tumor microenvironment and prognosis of prostate cancer

**DOI:** 10.1007/s12672-024-01006-z

**Published:** 2024-06-04

**Authors:** Tao Guo, Jian Wang, Xiangyu Meng, Ye Wang, Yihaoyun Lou, Jianglei Ma, Shuang Xu, Xiangyu Ni, Zongming Jia, Lichen Jin, Chengyu Wang, Qingyang Chen, Peng Li, Yuhua Huang, Shancheng Ren

**Affiliations:** 1 Department of Urology, Changzheng Hospital, Naval Medical University, Shanghai, China; 2 Department of Urology, The First Affiliated Hospital of Soochow University, Suzhou, China; 3 Department of Urology, Affiliated Hospital of Jiangnan University, Wuxi, China; 4 Department of Urology, The First Affiliated Hospital of Nanjing Medical University, Nanjing, China; 5 Department of Gastroenterology, The First Affiliated Hospital of Soochow University, Suzhou, China; 6 Department of Urology, Huzhou Central Hospital, Affiliated Central Hospital Huzhou University, Huzhou, China

**Keywords:** Zinc homeostasis, Tumor microenvironment, Prognosis, Prostate cancer, MT1A

## Abstract

**Background:**

Dysregulation of zinc homeostasis is widely recognized as a hallmark feature of prostate cancer (PCa) based on the compelling clinical and experimental evidence. Nevertheless, the implications of zinc dyshomeostasis in PCa remains largely unexplored.

**Methods:**

In this research, the zinc homeostasis pattern subtype (ZHPS) was constructed according to the profile of zinc homeostasis genes. The identified subtypes were assessed for their immune functions, mutational landscapes, biological peculiarities and drug susceptibility. Subsequently, we developed the optimal signature, known as the zinc homeostasis-related risk score (ZHRRS), using the approach won out in multifariously machine learning algorithms. Eventually, clinical specimens, Bayesian network inference and single-cell sequencing were used to excavate the underlying mechanisms of MT1A in PCa.

**Results:**

The zinc dyshomeostasis subgroup, ZHPS2, possessed a markedly worse prognosis than ZHPS1. Moreover, ZHPS2 demonstrated a more conspicuous genomic instability and better therapeutic responses to docetaxel and olaparib than ZHPS1. Compared with traditional clinicopathological characteristics and 35 published signatures, ZHRRS displayed a significantly improved accuracy in prognosis prediction. The diagnostic value of MT1A in PCa was substantiated through analysis of clinical samples. Additionally, we inferred and established the regulatory network of MT1A to elucidate its biological mechanisms.

**Conclusions:**

The ZHPS classifier and ZHRRS model hold great potential as clinical applications for improving outcomes of PCa patients.

**Supplementary Information:**

The online version contains supplementary material available at 10.1007/s12672-024-01006-z.

## Introduction

Prostate cancer (PCa) is the most frequently diagnosed cancer in men, and it currently has the second-highest survival rate among patients [1]. Due to the central role of the androgen receptor (AR) in the progression of PCa, androgen deprivation therapy (ADT) is initially effective in most patients [2, 3]. Nevertheless, the eventual development to the aggressive castration-resistant prostate cancer (CRPC) is unavoidable [4]. Despite the application of new-generation AR-targeted drugs, the drug-resistance remains a universal issue [5]. Thus, it is imminent to research the new diagnostic and therapeutic approaches to PCa [6–9].

As the second most abundant trace element in the body, zinc plays vital roles in the cellular growth and immune system, including the prostate [10]. High levels of zinc are detected in the prostate organ, and the concentration of zinc in the prostate fluid is much higher than that in the plasma [11]. The primary function of zinc accumulation in prostate epithelial cells is to inhibition of mitochondrial aconitase, leading to citrate production [12]. Zinc transporters and metallothioneins (MTs) function as regulators of zinc homeostasis [13]. In mammalian cells, two key zinc transporter families, ZIP (SLC39) and ZnT (SLC30), are responsible for the zinc influx and efflux, respectively [14], and MTs bind zinc ions through their cysteine-rich domain to regulate the distribution, storage and release of zinc [15]. The coordinated activities of zinc transporters and MTs form the biological basis for zinc homeostasis.

Consistently, compelling studies have reported a considerable decline in zinc concentration in PCa, relative to that in normal tissue [16]. Zinc dysregulation in prostatic intraepithelial neoplasia and prostate adenocarcinoma is strongly linked to ZIP1 downregulation [17]. Other altered zinc transporters that may be involved include ZIP2, ZIP3, ZIP4, and ZnT4 [18–20]. Attenuated MT1 and MT2 expressions are also observed in prostate tumors [21]. Insufficient zinc accumulation leads to the continuation of TCA cycle, establishing an energy-efficient environment for malignant cells [22]. An additional troublesome consequence of disordered zinc homeostasis is the defect in the antioxidant defense system [23]. Excessive oxidation induces DNA damage, raising the likelihood of cancer initiation and development. Moreover, zinc dyshomeostasis is accompanied by an altered tumor microenvironment (TME), impairing both innate and adaptive immunity and sculpting them to be pro-tumorigenic [24]. However, mechanisms by which dysregulated zinc homeostasis can impact PCa are still poorly understood.

To address this issue, we attempted to develop subtype stratifications based on profiles of zinc homeostasis regulators. The heterogeneity between subtypes in terms of immune, mutation, and clinical was unraveled to facilitate the understanding of PCa from the perspective of zinc disorder. Other than that, a reliable risk signature was developed and verified in multiple cohorts. The risk-stratification model will potentially benefit clinical diagnosis and treatment. Finally, we identified a prominent tumor-suppressor gene, MT1A, that can serve as a valuable biomarker and explored its regulatory network.

## Materials and methods

### Acquisition and processing of data

The Cancer Genome Atlas (TCGA, http://portal.gdc.cancer.gov/), Gene Expression Omnibus (GEO, https://www.ncbi.nlm.nih.gov/geo/), cBioPortal (https://www.cbioportal.org/) and University of California, Santa Cruz Xena (UCSC Xena, https://xena.ucsc.edu/) online databases served as the sources for our study. For RNA sequencing datasets (TCGA-Pancancer and DKFZ2018), the profiles were normalized as transcripts per kilobase million, and then log_2_(x + 1) transformed. Scale normalization (*limma* package) and the same logarithmic transformation were applied to array datasets (MSKCC2010, GSE70768, GSE70769, and GSE116918). UCSC Xena also provided mutation and methylation data. The abbreviations and full names of all cancers included in the pan-cancer analysis are listed in Supplementary Table 1. We obtained a series of tumor sequencing scores, including tumor mutational burden (TMB), microsatellite instability (MSI), homologous recombination deficiency (HRD), loss of heterozygosity (LOH), DNA methylation-based stemness score (DNAss), RNA expression-based stemness score (RNAss), differentially methylated probes-based stemness score (DMPss), and enhancer elements/DNA methylation-based stemness score (ENHss) from the Sangerbox platform (http://vip.sangerbox.com/) [25]. The calculation of immune, stromal, and estimate scores were calculated using the *estimate* package.

In the processing of single-cell RNA sequencing data, the standard Seurat workflow was implemented using the *Seurat* package. We integrated data from different samples with the iterative clustering method (*harmony* package) [26]. Cells that conform to the following criteria were preserved: (1) the number of expressed genes ranged from 600 to 5000; (2) the proportion of mitochondrial genes was less than 15%; and (3) the percentage of hemoglobin genes was less than 3%.

To identify the genes involved in zinc homeostasis, we searched The Molecular Signatures Database (MSigDb, https://www.gsea-msigdb.org/gsea/msigdb), and obtained five related gene sets (Supplementary Table 2). A total of 51 genes were incorporated ultimately (Supplementary Table 3).

### Consensus clustering

According to the zinc homeostasis genes associated with prognosis, consensus clustering (*ConsensusClusterPlus* package) was performed to discover new subtypes in the TCGA-PRAD cohort. For clustering, the partitioning around medoids (PAM) clustering approach by 1—Pearson correlation, and 500 iterations were the applied parameters. The optimal number of clusters was determined by the inter-sample correlation coefficient matrix, cumulative distribution function (CDF) curve, and proportion of ambiguous clustering (PAC) statistic [27]. Subsequently, principal component analysis (PCA) and uniform manifold approximation and projection (UMAP) plots depicted the clustering results.

### Nearest template prediction (NTP) validation

The subtype discrimination in other cohorts was based on the NTP-based classifier (*CMScaller* package) [28]. We identified the subtype-specific genes across subtypes using the PAM approach, and selected them as the characteristic signature. The NTP algorithm used these signature genes to predict the subgroups in each test dataset.

### Cell infiltration assessment

We assessed the relative abundances of cell infiltrations using six algorithms, including TIMER, CIBERSORT, quanTIseq, MCP-counter, xCell and EPIC [29–34]. Meanwhile, based on previously reported cell markers [35], single sample gene set enrichment analysis (ssGSEA) was applied to infer the abundance of 28 immune cell types (*GSVA* package).

### Genomic alteration spectrums

To comprehensively interrogate differences in genomic mutation between subtypes, we analyzed somatic mutation and copy number variation (CNV) data. The single nucleotide polymorphism (SNP) landscapes were visualized using the *maftools* package [36]. We summarized the affected oncogenic pathways, and drugs targeting mutant oncoproteins. Meanwhile, the degree of CNV was quantified by Genomic Identification of Significant Targets in Cancer 2.0 (GISTIC 2.0) [37], and we compared the burdens of CNV at the arm and focal levels.

### Assessment of potential biological functions

The identification of differentially expressed genes (DEGs) in bulk sequencing data was determined using the *limma* package, and in single-cell data, we used the function “FindMarkers” (*Seurat* package). In the next step, we selected the DEGs for over-representation and Proteomaps analyses (https://proteomaps.net/). The detected DEGs were input into ClueGO (the Cytoscape plug-in) for enrichment analysis [38]. Based on the ranked list of genes, gene set enrichment analysis (GSEA) was run via the *clusterProfiler* package [39]. In addition, to describe the strength of the underlying biological functions, we used gene set variation analysis (GSVA) to score each term (*GSVA* package) [40]. The functional interpretation of module genes was performed using Metascape (https://metascape.org/gp/index.html) [41]. All available gene sets, including Gene Ontology (GO), Kyoto Encyclopedia of Genes and Genomes (KEGG), Hallmark and Reactome, were downloaded from MSigDb.

### Prediction of drug susceptibility

The predictive process involves calculating the half-maximal inhibitory concentration (IC50) to evaluate the therapeutic agent sensitivity of each patient (*oncoPredict* package) [42], The training set for prediction was obtained from the Cancer Therapeutics Response Portal (CTRP) database, which contains information on drug responses [43]. The Gene Set Cancer Analysis (GSCA, http://bioinfo.life.hust.edu.cn/GSCA/#/) platform provided detailed information on the associations between the imported genes and drugs, based on the Genomics of Drug Sensitivity in Cancer (GDSC) resource [44, 45].

### Weighted gene co-expression network analysis (WGCNA)

In order to generate network modules of co-expressed transcripts, WGCNA was implemented using the *WGCNA* package [46]. We estimated the appropriate soft-thresholding power (β), following the scale-free network property. The highly correlated module for the trait was recognized and selected for further analysis. However, since the gray module was composed of unclustered genes, it was excluded from subsequent research.

### Construction of the prognostic signature based on machine learning algorithms

A total of 10 machine learning algorithms were used to establish the highly accurate and stable model. These algorithms included the elastic network (Enet), least absolute shrinkage and selection operator (Lasso), Ridge, CoxBoost, supervised principal components (SuperPC), survival support vector machine (Survival-SVM), stepwise Cox (StepCox), random survival forest (RSF), partial least squares regression for Cox (plsRcox) and generalized boosted regression (GBM) (*glmnet*, *CoxBoost*, *superpc*, *survivalsvm*, *survival*, *randomForestSRC*, *plsRcox*, and *gbm* packages). The first step in generating the signature involved screening for prognosis-related genes, with the univariate Cox regression analysis (*P* < 0.05). Then, based on the screened genes, we used the aforementioned machine learning methods to build different models. TCGA-PRAD dataset served as a training dataset for model construction, whereas the other cohorts were applied as validation datasets. The Harrell concordance index (C-index) of each signature was calculated across TCGA-PRAD, MSKCC2010, and GSE70768. After the comparison, the optimal model with the highest average C-index was the final selected signature. The median score in the obtained signature was used as the threshold for categorizing patients into high- and low-score groups.

### Protein–protein interaction (PPI) networks

For the PPI analysis, we incorporated two databases for searching the possible contacts. The Compartmentalized Protein–Protein Interaction (ComPPI, https://comppi.linkgroup.hu/) database provided lists of interacting proteins, interaction scores and subcellular localizations [47]. Moreover, another network was constructed by GeneMANIA (https://genemania.org/) [48].

### Gene regulatory network (GRN) inference

Since simple correlations alone cannot demonstrate the directions of regulation among different genes, therefore we inferred directed edges using the Bayesian network (BN) based on TCGA-PRAD expression data (*CBNplot* package) [49]. DEGs between high and low MT1A expression groups were initially detected, and functional annotation was performed through GO enrichment. After that, the "Response to metal ions" pathway containing MT1A was selected for BN analysis.

### Immunohistochemical (IHC) staining

A total of 10 pairs of cancerous and paraneoplastic tissues were harvested from Department of Urology, The First Affiliated Hospital of Soochow University. Subsequently, we performed IHC and evaluated immunoreactive score (IRS) on each section. The primary antibody for MT1A (NBP1-97493; 1:150 dilution) was obtained from Novus Biologicals.

### Statistical analysis

R 4.2.2 software was responsible for all data analysis and visualization. When comparing continuous variables, we used the t-test or Wilcoxon rank-sum test. Chi-square or Fisher exact tests were the statistical method applied in comparing categorical data. Pearson’s correlation analysis was employed to assess the correlation between two continuous variables. The time-dependent area under the curve (AUC) was calculated using the *timeROC* package. Survival analyses include the Cox proportional hazard model and Kaplan–Meier (KM) analysis (*survival* and *survminer* packages). The outcome measures for prognosis were overall survival (OS), disease-specific survival (DSS), progression-free interval (PFI), disease–free survival (DFS) and biochemical recurrence (BCR). All statistical tests were two-sided, and *P* < 0.05 was considered to indicate statistically significant difference.

## Results

### Dysregulated zinc homeostasis-related genes (ZHRGs) across the pan-cancer atlas

**Fig. 1:**
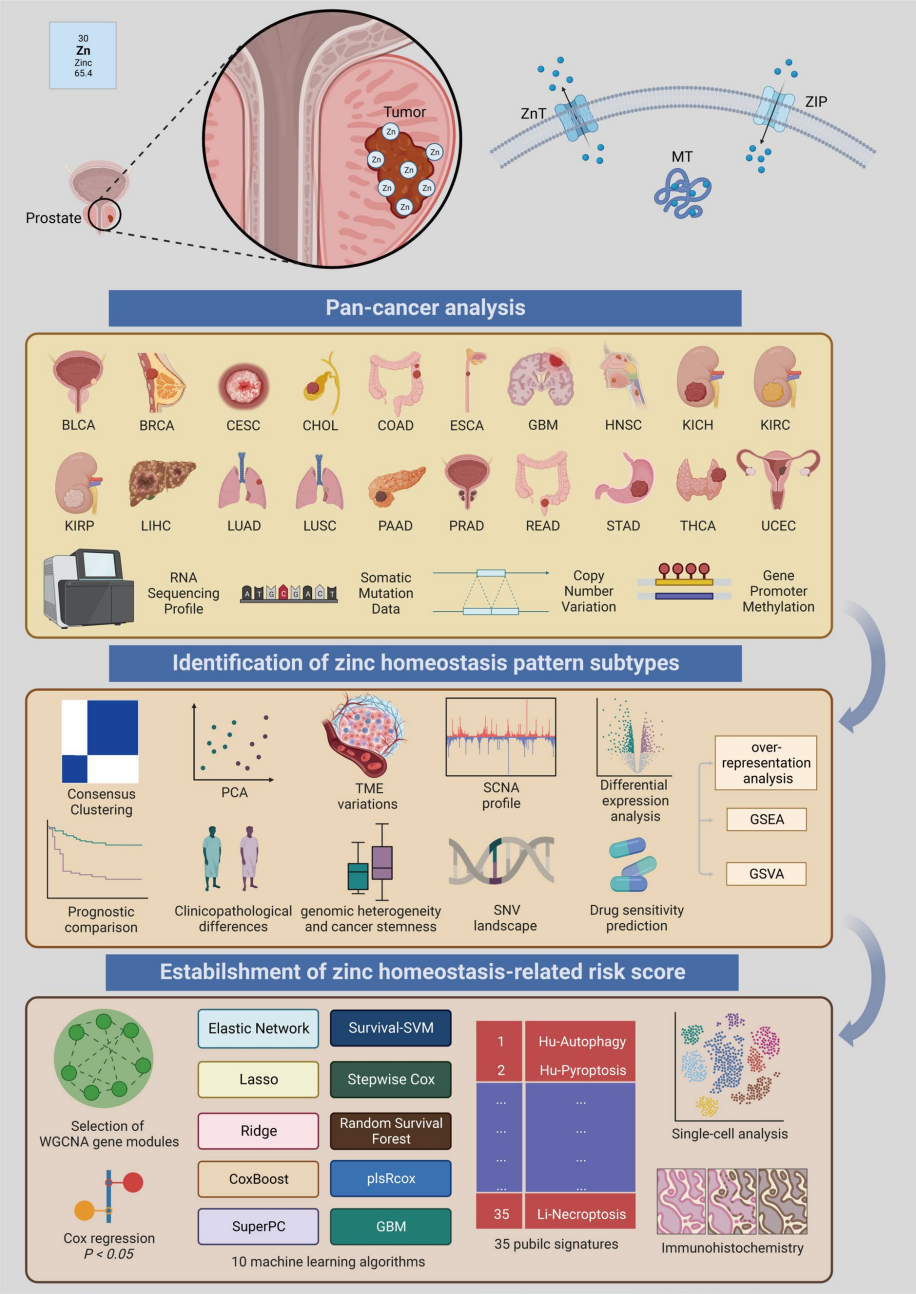
Flow diagram illustrating the overall research process. The image was generated from BioRender (https://biorender.com/)

**Fig. 2:**
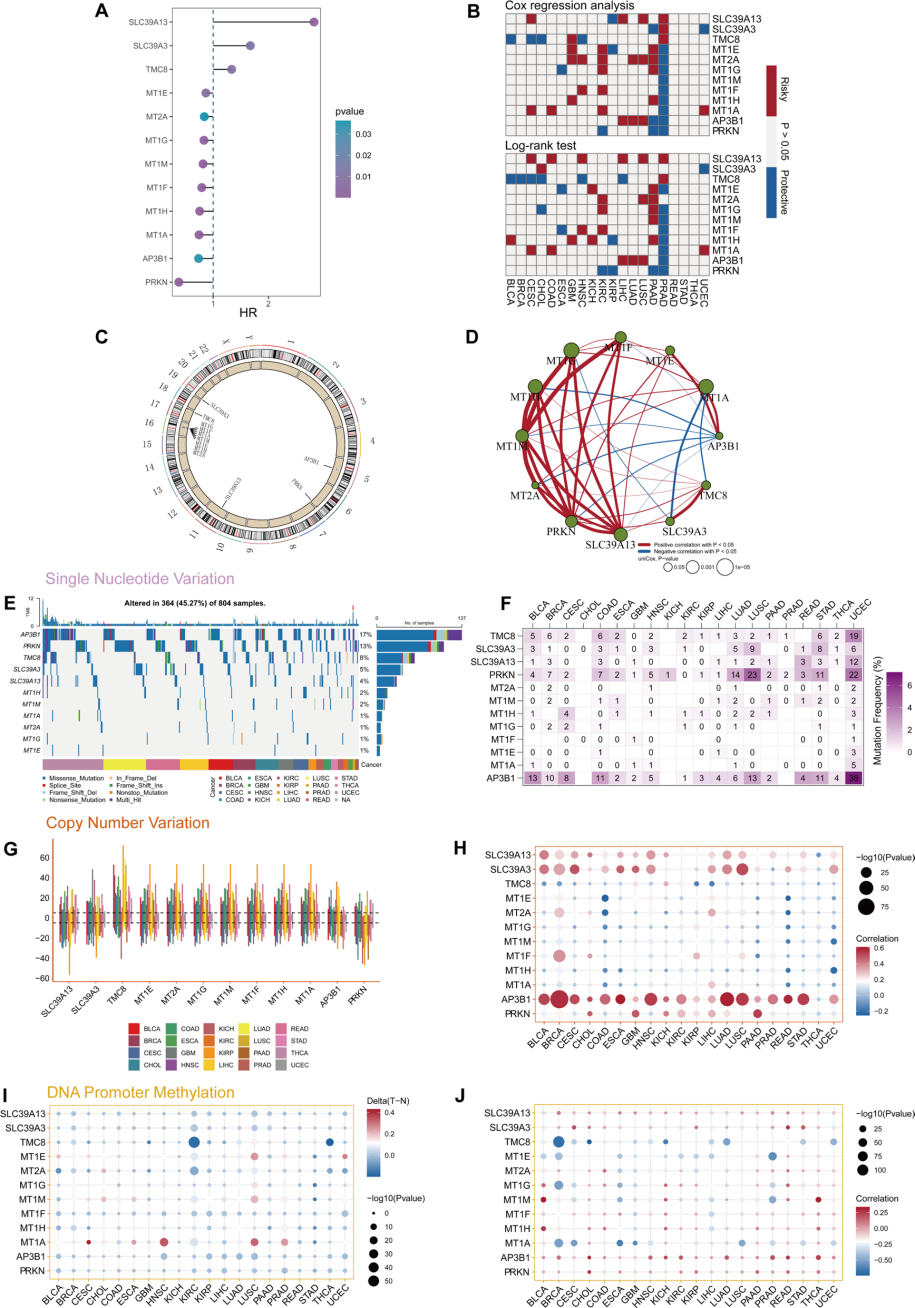
Multi-omics analysis of zinc homeostasis regulators at the pan-cancer level. **A** Identification of DFS-related ZHRGs in TCGA-PRAD. **B** Cox regression analysis and log-rank test on DFS in different cancers. **C** Genomic positions of ZHRGs. **D** Association network based on the expression of ZHRGs. **E** Oncoplot displaying the SNV profiles of ZHRGs. **F** Mutational frequencies of ZHRGs in 20 tumors. **G** CNV alterations of each ZHRG. **H** Associations between the expression of ZHRGs and CNV alterations. **I** Differences in promoter methylation levels between tumor and normal samples. **J** Correlations between the expression of ZHRGs and promoter methylation levels

The flowchart of the research is presented in Fig. 1. We first synthesized the zinc homeostasis index (ZHI) based on the collected gene list using ssGSEA. ZHI was observed to be decreased in most cancers compared with that in the normal adjacent samples, including THCA, STAD, READ, PRAD, LUSC, LUAD, LIHC, KIRP, KIRC, KICH, ESCA, COAD, and CHOL (Supplementary Fig. 1). We then identified the following DFS-related ZHRGs in TCGA-PRAD: SLC39A13, SLC39A3, TMC8, MT1E, MT2A, MT1G, MT1M, MT1F, MT1H, MT1A, AP3B1, and PRKN (Fig. 2A and Supplementary Table 4). These ZHRGs exhibited inconsistent prognostic roles across different cancers, indicating the diversity of their functions (Fig. 2B). All ZHRGs in the metallothionein family were concentrated on chromosome 16, while the remaining ZHRGs were scattered on other chromosomes (Fig. 2C). Overall, there were positive inter-correlations between ZHRG expressions (Fig. 2D).

Among the twelve ZHRGs, AP3B1 had the highest SNP frequency, followed by PRKN (Fig. 2E). The incidences of ZHRG SNPs in PRAD were within a considerably low mutation range (Fig. 2F). To further explore the genetic alterations of ZHRGs in pan-cancer, we plotted the CNV frequency profiles (Fig. 2G). TMC5 was highly prone to copy-number gains, whereas PRKN was primarily characterized by copy-number deletions. Additionally, heterozygosity deletions appeared to be common in PRAD (Supplementary Fig. 2). The CNV patterns were highly similar among the metallothionein family, probably owing to their shared chromosomal loci. The expression of SLC39A13, SLC39A3, AP3B1, and PRKN were positively correlated with CNVs (Fig. 2H). The ZHRGs exhibited similar promoter methylation patterns, except for MT1A (Fig. 2I). In CESC, HNSC, LUSC and PRAD, MT1A exhibited hypermethylation. We also observed complex associations between ZHRG expression and promoter methylation levels (Fig. 2J). Notably, SLC39A13 expression was significantly and positively correlated with IC50 values of QL-XI-92, BHG712, BIX02189, THZ-2–429, TL-1–85, and DMOG, suggesting a potential role of SLC39A13 in promoting drug resistance (Supplementary Fig. 3).

### Subtype identification through consensus clustering

**Fig. 3:**
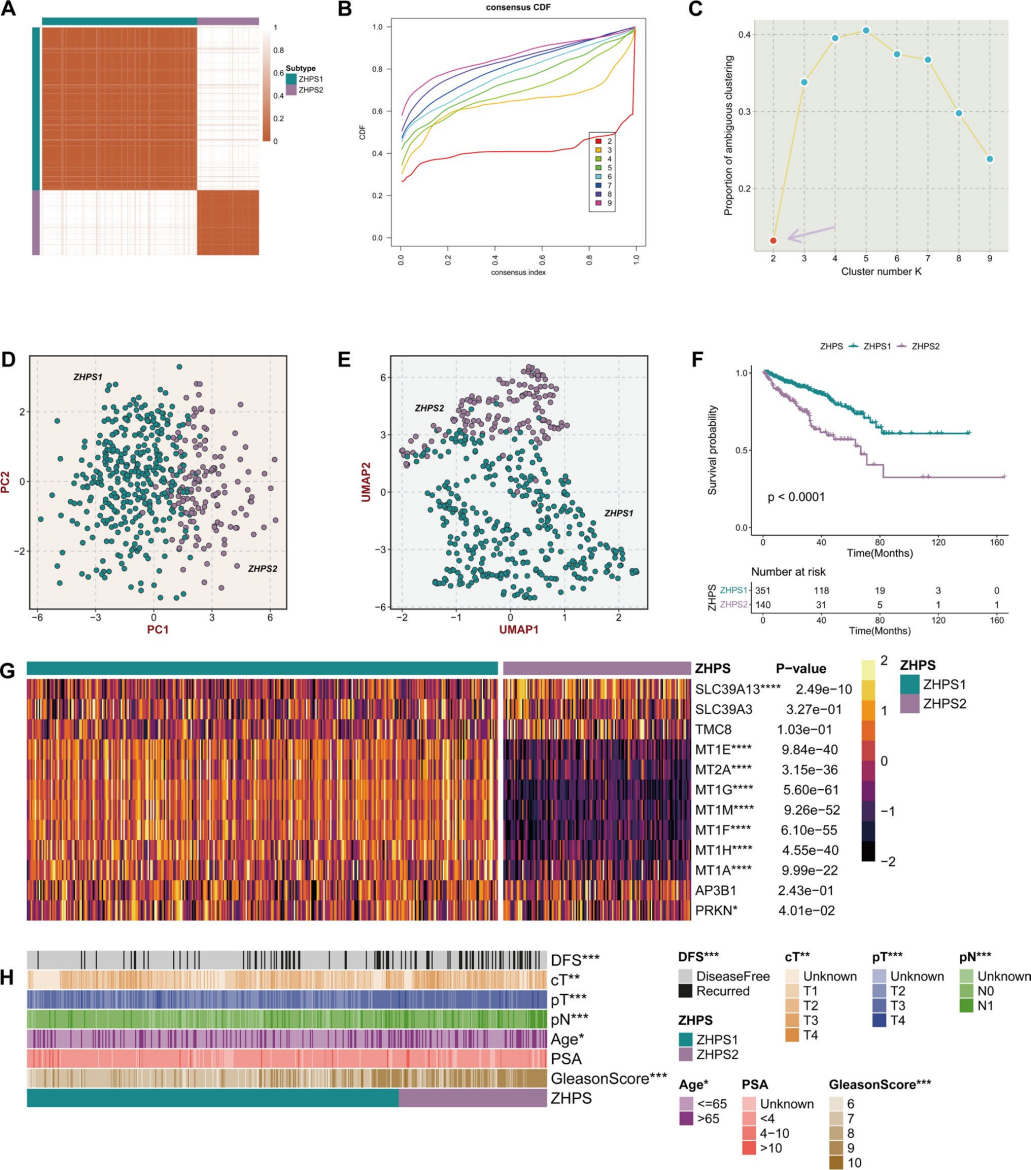
Identification of two distinct subtypes with heterogeneous ZHRG expression**. A** Consensus map of clustering results in TCGA-PRAD. **B** Cumulative distribution function curves, with cluster number k set from 2 to 9. **C** Proportion of ambiguous clustering (PAC) score, and the minimal ambiguity depicting the optimal number k was 2. **D** Principal component plot showing patients in two ZHPSs. **E** Uniform manifold approximation and projection plot analysis showing the distribution of two subtypes. **F** Kaplan–Meier curves of DFS between ZHPS1 and ZHPS2. **G** Heatmap depicting the expression of ZHRGs in the two subtypes. **H** Comparison of clinical and pathological characteristics. Significance levels are denoted by the following symbols: *P < 0.05; **P < 0.01; ***P < 0.001; ****P < 0.0001

Multiple lines of evidence supported that the optimal number of clusters for consistent clustering based on 12 ZHRGs was two (Fig. 3A, B, and C). Both PCA and UMAP plots revealed distinct distributions of two subgroups, zinc homeostasis pattern subtype 1 (ZHPS1) and zinc homeostasis pattern subtype 2 (ZHPS2), validating the rationality of the clustering (Fig. 3D, E). As illustrated in Fig. 3F, ZHPS2 possessed a worse prognosis relative to ZHPS1. This observation was consistent with the desert of good-prognosis genes (MT1E, MT2A, MT1G, MT1M, MT1F, MT1H, and MT1A) in ZHPS2 (Fig. 3G). Furthermore, ZHPS2 held more patients in advanced stages (clinical T, pathologic T and pathologic N stages) (Fig. 3H).

### Profiles of immune cellular composition and function

**Fig. 4:**
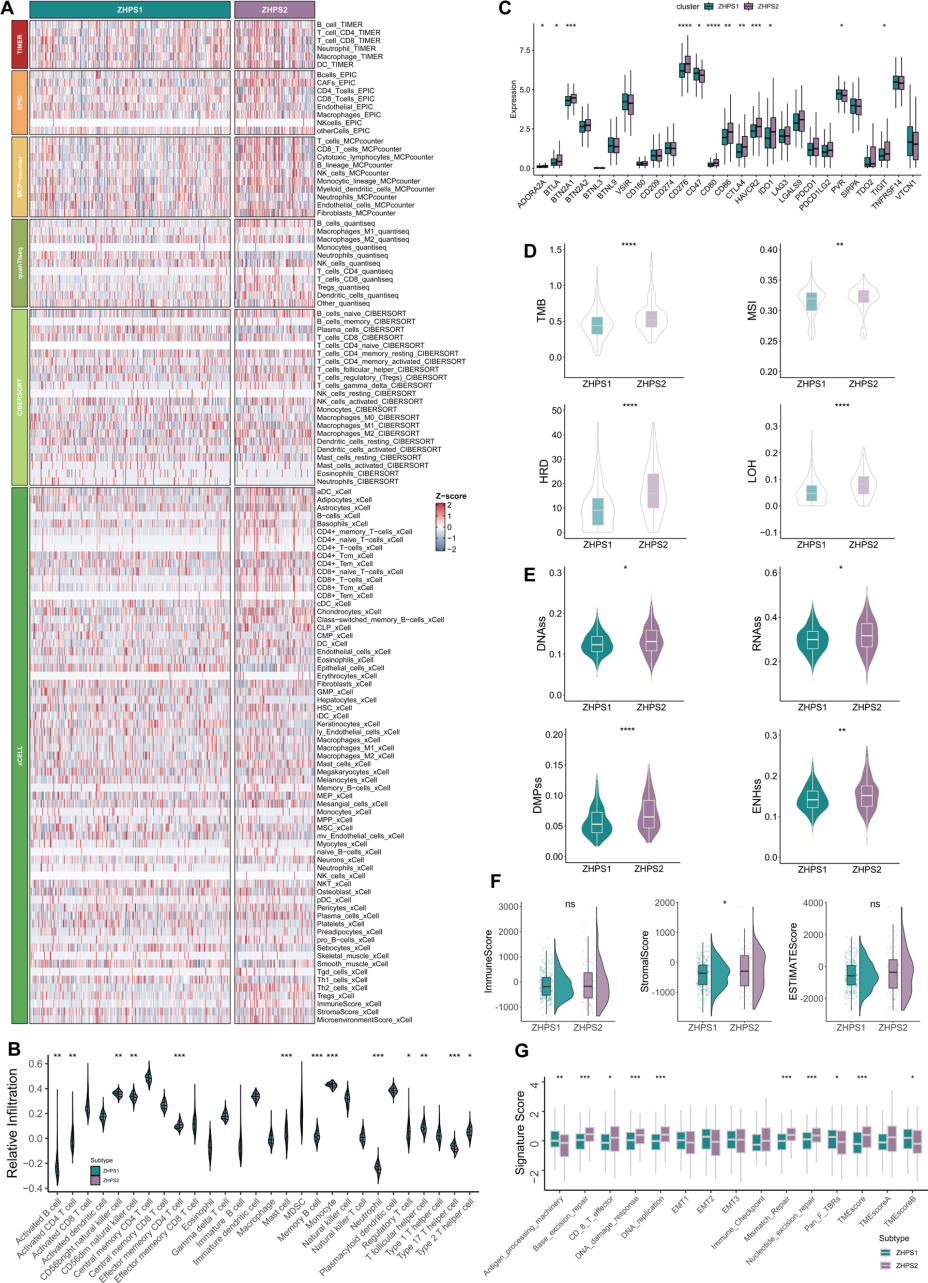
Investigation of immune profiles. **A** Estimation of immune infiltrations using six methods (TIMER, CIBERSORT, quanTIseq, MCP-counter, xCell, and EPIC). **B** ssGSEA results revealing differences in immune cell abundances **C-G** Immune landscapes between the two subtypes: **C** immune checkpoints, **D** genomic heterogeneity indices (TBM, MSI, HRD and LOH), **E** stemness indices (DNAss, RNAss, DMPss and ENHss), **F** scores derived from the ESTIMATE algorithm (immune score, stromal score, and estimate score) and **G** immune-related pathways. Significance levels are denoted by the following symbols: *P < 0.05; **P < 0.01; ***P < 0.001; ****P < 0.0001

The holistic immune cell infiltration levels in ZHPS1 and ZHPS2 were calculated by multiple algorithms (Fig. 4A, B). As regulators of immune homeostasis, immune checkpoints showed higher expression in ZHPS2 (e.g., ADORA2A, BTLA, BTN2A1, CD276, CD80, CD86, CTLA4, HAVCR2, IDO1 and TIGIT), thereby weakening antitumor immunity (Fig. 4C). When comparing genomic heterogeneity indices, we found that TBM, MSI, HRD, and LOH values were relatively high in ZHPS2 (Fig. 4D). The distributions of the stemness indices (DNAss, RNAss, DMPss and ENHss) were similar between the subtypes (Fig. 4E). Therefore, we inferred that ZHPS2 may be a more suitable subgroup for immune checkpoint blockades. The immune score did not differ between the two subtypes. Conversely, the stromal score was comparatively lower in ZHPS1 (Fig. 4F). Furthermore, ZHPS1 demonstrated a higher capability for antigen processing (Fig. 4G).

### Variances in mutational landscapes

**Fig. 5:**
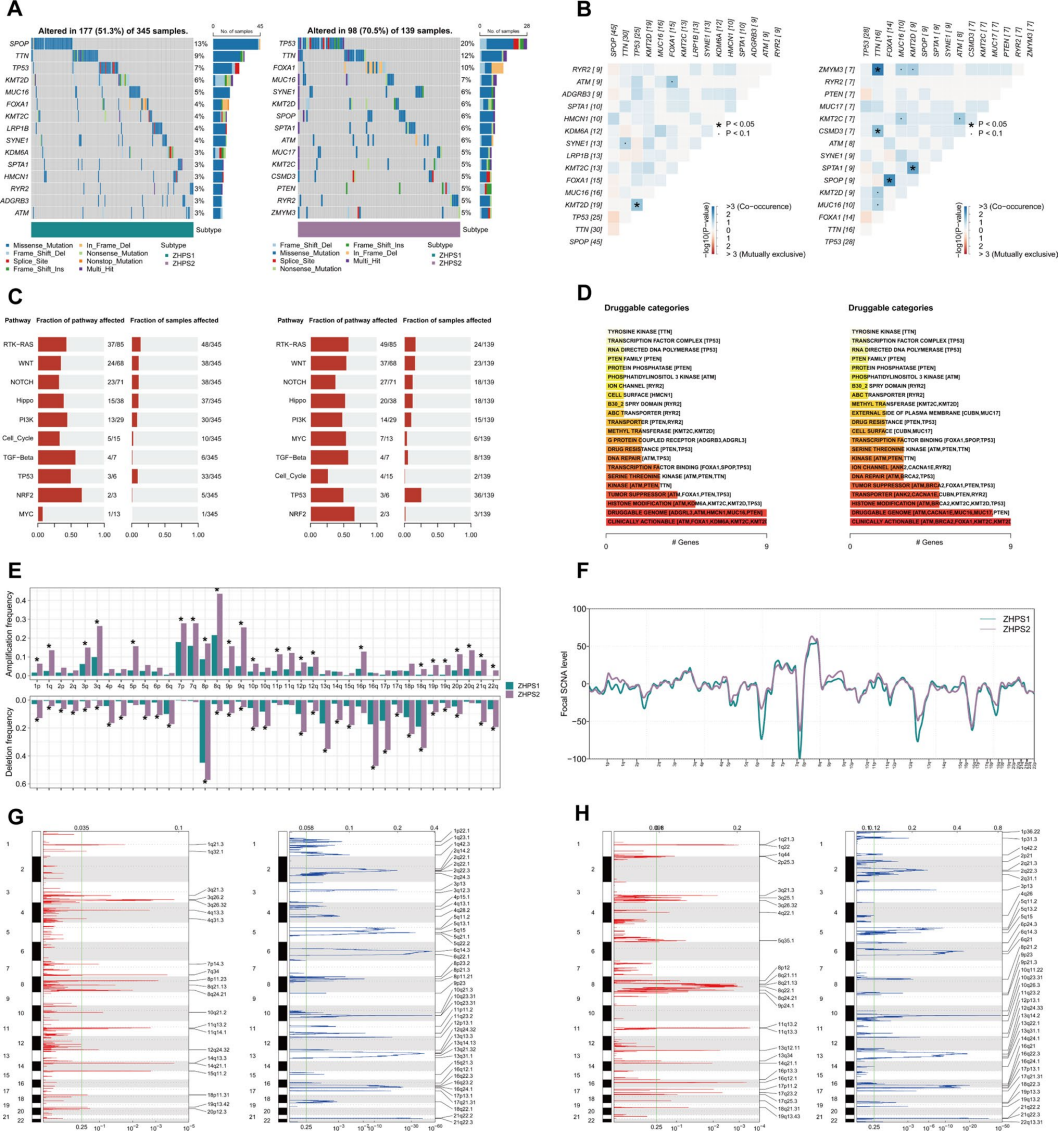
Comprehensive analysis of genomic variations. **A** Oncoplots of ZHPS1 and ZHPS2. **B** Co-occurrence and exclusivity of the top mutated genes in the two subtypes. **C** Signaling pathways affected by mutations. **D** Potentially druggable categories from different subtypes. **E–F** Amplifications and deletions at **E** the arm-level and **F** focal-level. **G-H** Specific CNV gains and losses in **G** ZHPS1 and **H** ZHPS2

Taken altogether, the incidence of mutations was higher in ZHPS2 than in ZHPS1 (70.5% vs 51.3%) (Fig. 5A). Waterfall plots exhibited the 15 most frequently mutated genes for each subtype, with TP53, TTN, FOXA1, MUC16, SYNE1, KMT2D, SPOP, SPTA1, ATM, KMT2C, and RYR2 being present in both subtypes. Compared with ZHPS1, ZHPS2 had a higher frequency of mutations in TP53 (20% vs 7%), TTN (12% vs 9%), FOXA1 (10% vs 4%), MUC16 (7% vs 5%), SYNE1 (6% vs 4%), ATM (6% vs 3%), KMT2C (5% vs 4%) and RYR2 (5% vs 3%). Moreover, the overall mutation rates of ZHRGs in PRAD were considerably low, resulting in no significant differences between the two subtypes (Supplementary Fig. 4). Several distinct co-mutation patterns occurred (Fig. 5B). In ZHPS1, only TP53-KMT2D co-mutation was found, whereas in ZHPS2, co-mutations of TTN-ZMYM3, TTN-CSMD3, FOXA1-SPOP, and KMT2D-SPTA1 were observed. These mutated genes were primarily enriched in the RTK-RAS (49/85 vs 37/85), WNT (37/68 vs 24/68), NOTCH (27/71 vs 23/71), Hippo (20/38 vs 15/38), PI3K (14/29 vs 13/29) and MYC (7/13 vs 1/13) pathways, with higher fractions of affected pathways in ZHPS2 (Fig. 5C). Recently, the research on drugs targeting mutated proteins presents a scene in full swing. The main druggable category of the two subtypes was clinically actionable (Fig. 5D). However, the mutated genes contained slight differences: ZHPS1 exhibited mutations in ATM, FOXA1, KDM6A, KMT2C, and KMT2D, whereas ZHPS2 showed mutations in ATM, BRCA2, FOXA1, KMT2C, and KMT2D.

CNVs, which represent large-scale genomic instability, were integrated and compared. At the arm-level, the differences of CNV between the two subgroups were highly significant, manifested by the higher frequencies of amplification and deletion in ZHPS2 (Fig. 5E). In contrast, a similar distribution was found when comparing the focal-level CNVs (Fig. 5F). Patients in ZHPS1 were mainly amplified in the regions such as 3q26, 14q, and 7q34, and deleted in regions such as 6q14, 10q, and 13q (Fig. 5G). In ZHPS2, the primary amplified regions were 11q, 16q, and 8q, and deleted regions were 10q, 21q, and 16q (Fig. 5H).

### Investigation of the underlying mechanisms and drug sensitivity

**Fig. 6:**
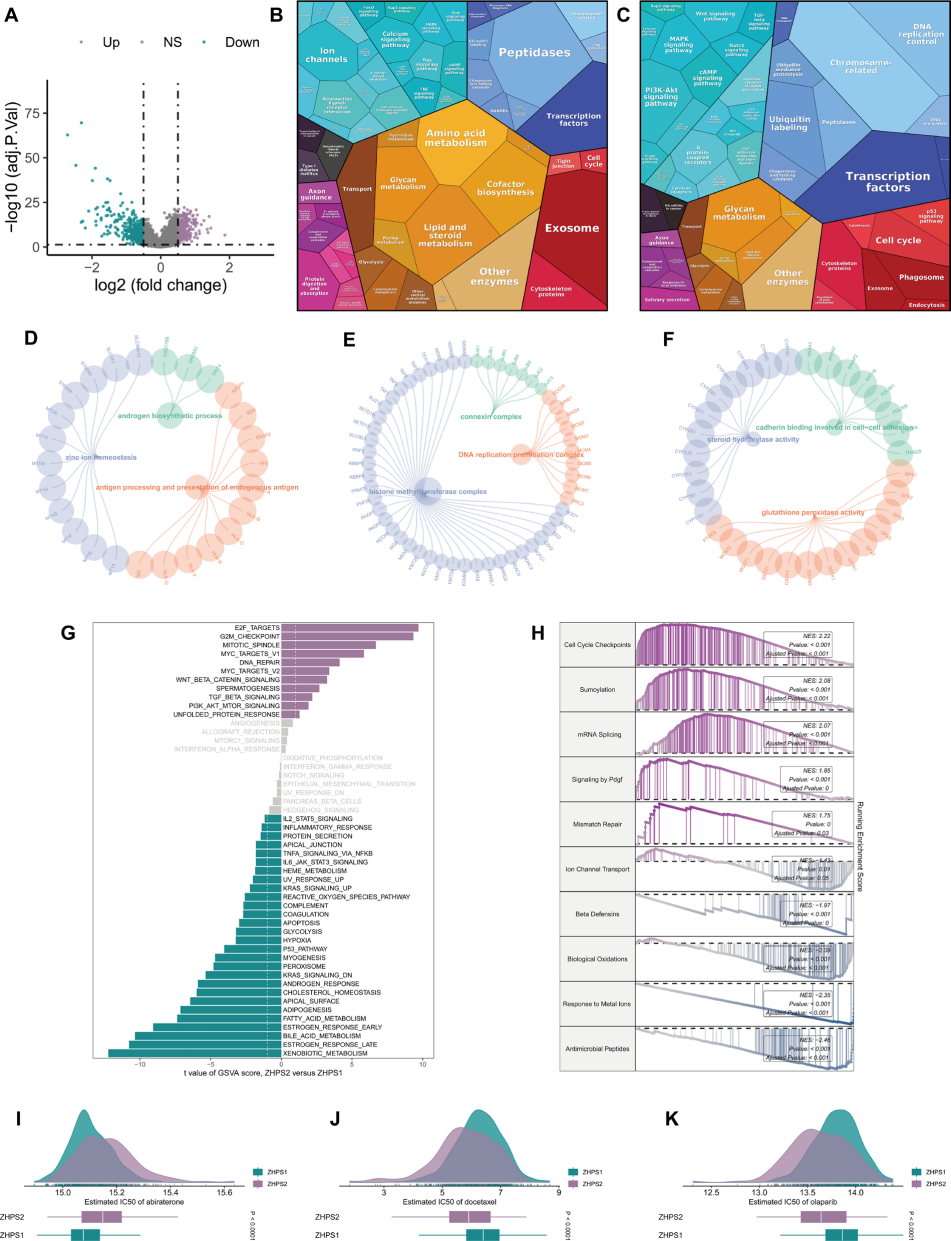
Biological peculiarities of the two subtypes. **A** Differential expression analysis for transcriptomes between the two subtypes. **B-C** Proteomaps demonstrating functional analysis in **B** ZHPS1 and **C** ZHPS2 based on the KEGG database. **D-F** GO enrichment analysis for **D** biological process, **E** cellular component and **F** molecular function. **G** Differences in Hallmark pathways through GSVA between ZHPS2 and ZHPS1. **H** GSEA of the Reactome pathways (ZHPS2 versus ZHPS1). **I-K** Estimated IC50 values of **I** abiraterone, **J** docetaxel and **K** olaparib

To understand the potential mechanisms contributing to the survival differences, we explored the variations in expression profiles between the subtypes (Fig. 6A). It was apparent that a large proportion of metabolically enriched terms was in ZHPS1, including amino acid, lipid, and steroid metabolism (Fig. 6B). Meanwhile, genetic information processing, which encompasses transcription factors and chromosome-related terms, emerged as was the main Proteomaps analysis module in ZHPS2 (Fig. 6C). We performed GO over-representation analysis of DEGs. The three terms with the highest enrichment score in biological process, cellular component, and molecular function were chosen for plotting. GO enrichment analysis revealed significant differences between the two subgroups in the androgen biosynthetic process, zinc ion homeostasis, antigen processing, and presentation of endogenous antigen, connexin complex, DNA replication preinitiation complex, histone methyltransferase complex, steroid hydroxylase activity, cadherin binding involved in cell–cell adhesion activity and glutathione peroxidase activity (Fig. 6D, E, and F).

As seen in the GSVA results, E2F targets and G2M checkpoints were significantly associated with ZHPS2. The pathways closely linked to ZHPS1 were xenobiotic metabolism and estrogen response late (Fig. 6G). We found upregulated Reactome pathways in ZHPS2 were cell cycle checkpoints, sumoylation, mRNA splicing, signaling by PDGF, and mismatch repair. For ZHPS1, the subgroup was primarily enriched in antimicrobial peptides, response to metal ions, biological oxidants, beta defensins, and ion channel transport (Fig. 6H). Furthermore, according to drug-susceptibility analysis, ZHPS1 displayed a superior response to abiraterone, whereas ZHPS2 might be more sensitive to docetaxel and olaparib (Fig. 6I, J, and K). Among DNA repair-related genes, the frequencies of BRCA2-mutation and ATM-mutation were higher in ZHPS2 than in ZHPS1 (Supplementary Fig. 5).

### Verification of subtype classification in external cohorts

**Fig. 7:**
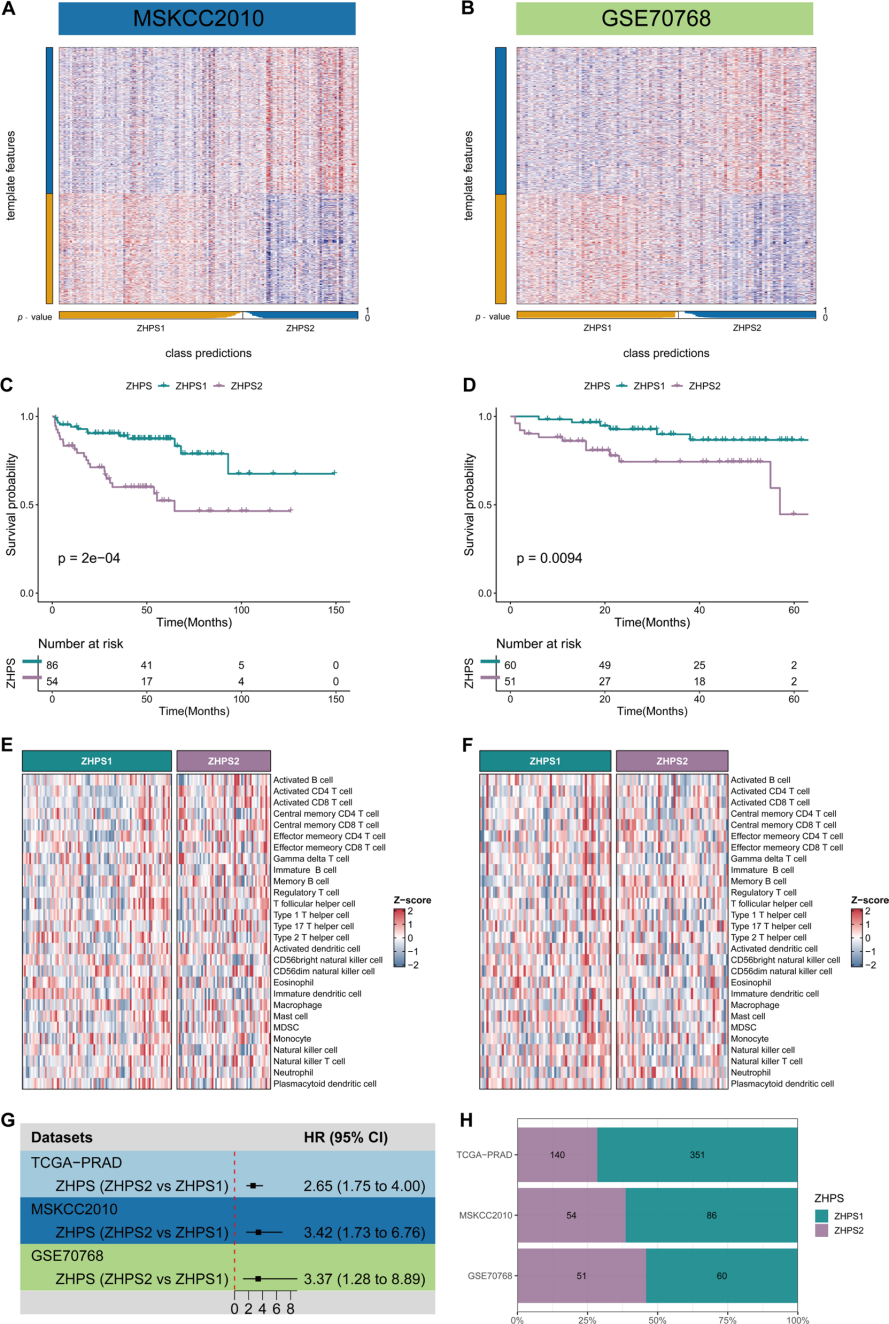
External validations of the classifier. **A-B** Heatmaps of the NTP classifier in **A** MSKCC2010 and **B** GSE70768. **C** Kaplan–Meier curves of DFS in MSKCC2010. **D** Kaplan–Meier curves of BCR in GSE70768. **E–F** Differences in immune cell infiltrations through ssGSEA in **E** MSKCC2010 and **F** GSE70768. **G** Univariate Cox regression of ZHPS (ZHPS2 versus ZHPS1) in TCGA-PRAD (DFS), MSKCC2010 (DFS) and GSE70768 (BCR). **H** Proportions of subtypes in each cohort

It was necessary not only to identify zinc homeostasis subtypes in TCGA-PRAD, but also to verify the reliability and stability of the classifier in external datasets. The signatures of each subtype in TCGA-PRAD were selected as input genes for the NTP classifier, and the subtypes of the validation sets were determined separately (Fig. 7A, B). KM analysis confirmed that ZHPS2 was associated with a more unfavorable prognosis in MSKCC2010 and GSE70768 (Fig. 7C, D). In both test sets, differences in the composition of TME between ZHPS classifications were not significant (Fig. 7E, F). Notably, ZHPS was competent as a prognostic predictor in univariate Cox regression analysis (Fig. 7G). From the ZHPS distribution of the different cohorts, we could find that the percentages of ZHPS1 were higher than those of ZHPS2 in all cohorts (Fig. 7H). Generally, ZHPS clustering was reproducible and stable in PCa.

### Multiple machine learning algorithms to build a robust signature

**Fig. 8:**
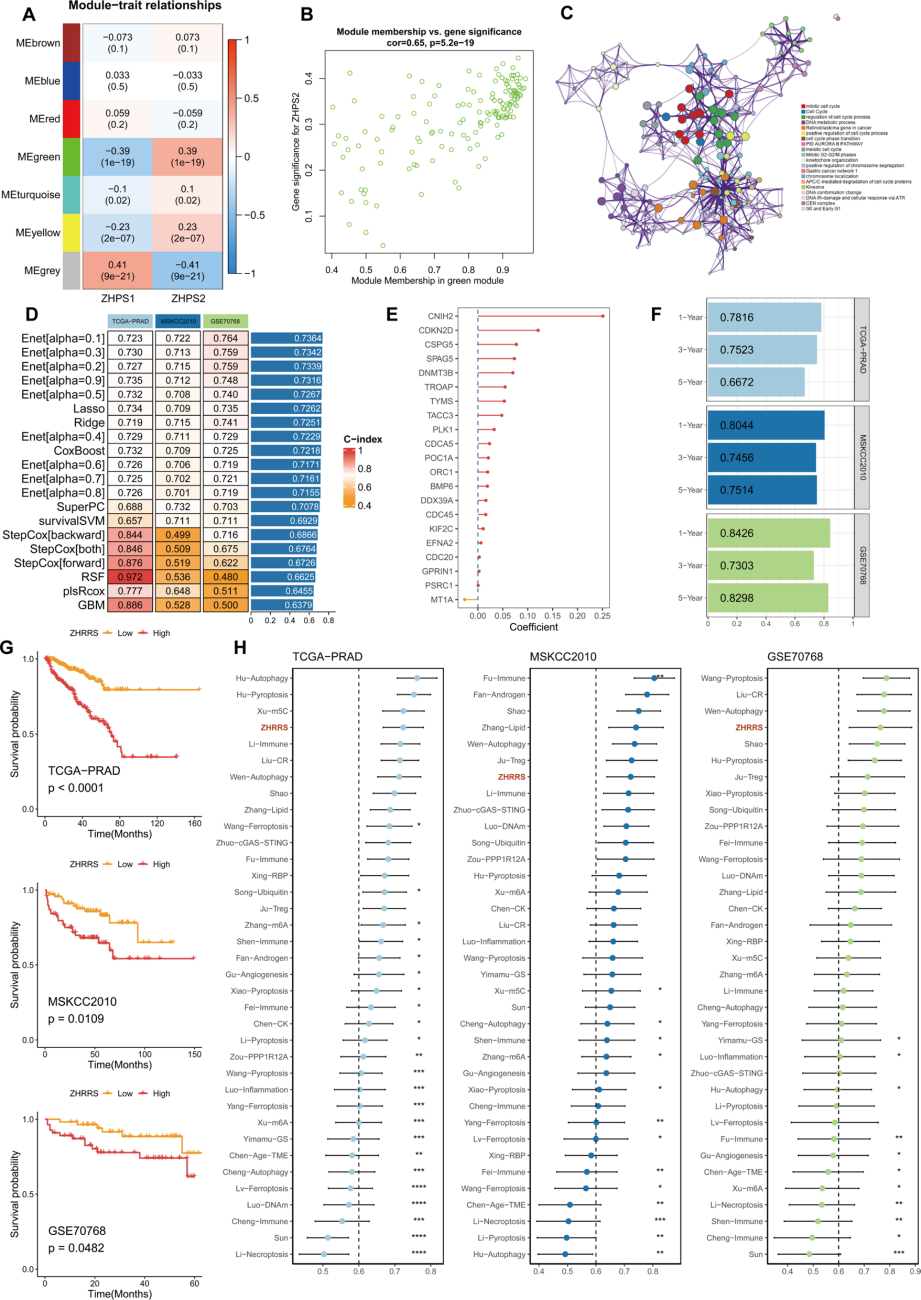
ZHRRS development and validation. **A** Correlations between each module and subtypes. **B** Correlation between gene significance and module membership in the green module. **C** Enrichment analysis based on genes from the green module using Metascape. **D** C-index generated by 10 machine learning algorithms under different datasets. **E** All coefficients in the signature derived from the elastic network (α = 0.1). **F** Time-dependent ROC AUCs in 1-, 3-, and 5-year time points. **G** Kaplan–Meier curves of DFS in TCGA-PRAD, MSKCC2010 and BCR in GSE70768. **H** C-index comparison of ZHRRS and 35 public signatures. Significance levels are denoted by the following symbols: *P < 0.05; **P < 0.01; ***P < 0.001; ****P < 0.0001

Aimed at discerning gene modules from the zinc homeostasis pattern, we set the soft threshold β to eight (no-scale R^2^ = 0.85) and followed the WGCNA procedure to construct a co-expression network (Supplementary Fig. 6). Except for the unclustered gray module, the green module showed the highest correlation with ZHPS2 in the module–trait relationships (Fig. 8A). In the green module, a statistically significant correlation of 0.65 was observed between gene significance and module membership (Fig. 8B), with 147 genes included (Supplementary Table 5). These genes exhibited strong correlations with cell cycle processes, such as the mitotic cell cycle, cell cycle, and regulation of cell cycle process (Fig. 8C).

Before developing the prognostic model, we carried out the univariate Cox regression analysis and identified 133 DFS-related genes (Supplementary Table 6). Among the various constructed models, the signature derived from the elastic network (α = 0.1) demonstrated the highest C-index (Fig. 8D). The final signature, referred to as the zinc homeostasis-related risk score (ZHRRS), comprised 21 constituent genes (Fig. 8E and Supplementary Table 7). The calculation formula was ZHRRS = ∑(Coef i * Expi). In other words, ZHRRS was the sum of the gene expression level multiplied by the corresponding coefficient. The discriminatory ability of ZHRRS was assessed using time-dependent receiver operating characteristic analysis (1-, 3- and 5-year AUCs: 0.7816, 0.7523 and 0.6672 in TCGA-PRAD; 0.8044, 0.7456 and 0.7514 in MSKCC2010; 0.8426, 0.7303 and 0.8298 in GSE70768) (Fig. 8F). In each cohort, the high-ZHRRS group exhibited a more dismal prognosis compared to the low-ZHRRS group (Fig. 8G). Simultaneously, the remarkable prognostic capability of ZHRRS was verified in the independent cohorts, including DKFZ2018, GSE70769, and GSE116918 (Supplementary Fig. 7). Numerous prognostic signatures based on next-generation sequencing or array technologies have emerged for PCa. Thus, a total of 35 published signatures were enrolled to compare the predictive performance with ZHRRS (Supplementary Table 8). Different risk scores were calculated based on the corresponding coefficient and gene expression. The C-index of each signature was calculated separately and finally compared. Notably, ZHRRS featured a superior performance among the published models (Fig. 8H), indicating its potential as an excellent prediction model.

### Advancements in the applications of ZHRRS

**Fig. 9:**
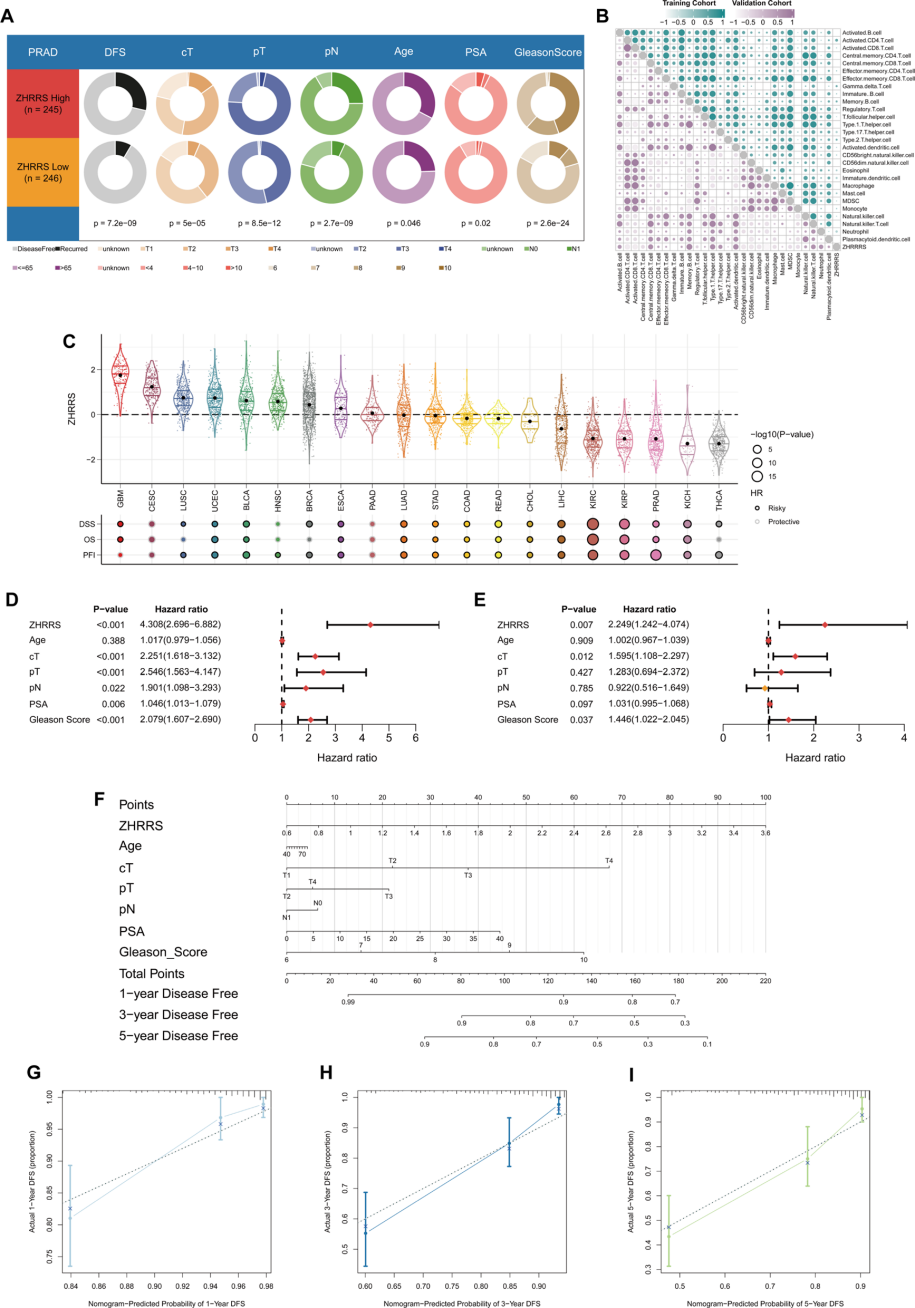
Clinical implications of ZHRRS for practical applications. **A** Different distributions of clinicopathologic features in ZHRRS-high group and ZHRRS-low group. **B** Correlations between ZHRRS and immune cell levels in the training and validation cohorts. **C** Prognostic values of ZHRRS in pan-tumor (clinical endpoints: DSS, OS, and PFI). **D-E** Associations between ZHRRS, clinicopathologic characteristics and DFS in the **D** univariable and **E** multivariable Cox analysis. **F** Integrated nomogram for PCa prognosis prediction. **G-I** Calibration curves of the established nomogram in **G** 1-, **H** 3-, and **I** 5-year

Distributions of clinicopathological features between high- and low-risk groups differed significantly. The high-ZHRRS group exhibited higher proportions of recurrences, clinical T3–4, pathological T3–4, pathological N1, PSA > 10 μg/L, and Gleason Score 8–10 than the low–ZHRRS group (Fig. 9A). ZHRRS was capable of accurately predicting different ZHPSs (Supplementary Fig. 8). In both the TCGA-PRAD and validation cohorts, cell infiltration analysis revealed a dramatically inverse correlation between ZHRRS and most immune cell infiltration levels (Fig. 9B). Interestingly, ZHRRS had not only shown excellent predictive performance in PRAD, but also exerted extrapolation potential in many other cancers, except for CESC and PAAD (Fig. 9C).

Consistently, ZHRRS was regarded as a valuable prognostic factor in univariate and multivariate Cox regression analyses (Fig. 9D, E). Based on ZHRRS, age, clinical T stage, pathological T stage, pathological N stage, PSA, and Gleason Score, we constructed a nomogram of the multivariable model for predicting 1-, 3-, and 5-year DFS (Fig. 9F). Consequently, the nomogram-predicted DFS showed good consistency with the observed DFS (Fig. 9G, H, and I).

### Substantial roles of MT1A in PRAD

**Fig. 10:**
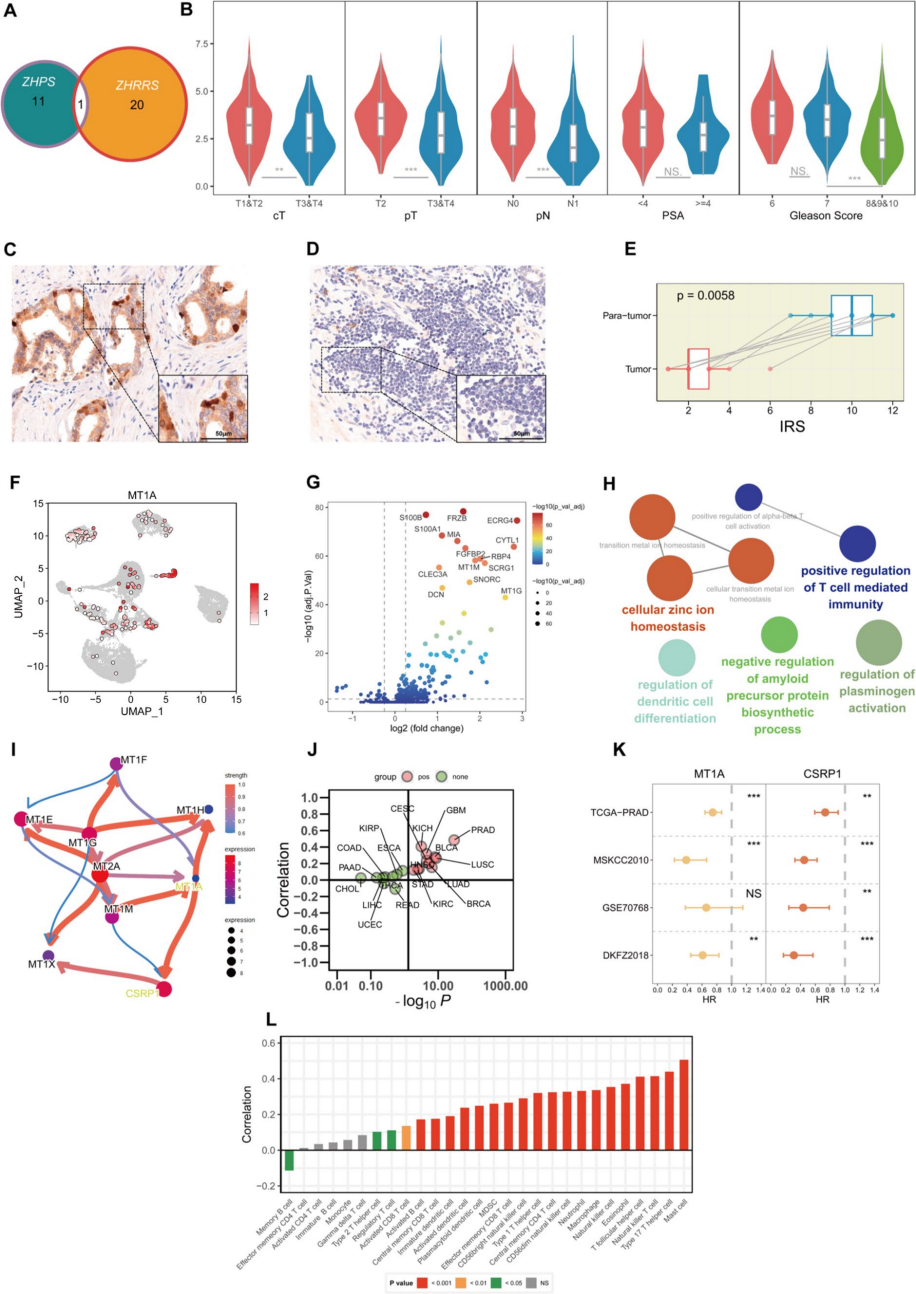
Core impacts of MT1A on PCa **A** MT1A is the only common gene between ZHPS and ZHRRS. **B** MT1A expression in different cT stages, pT stages, pN stages, PSA concentrations, and Gleason scores. **C-D** Immunohistochemical staining of MT1A in **C** paraneoplastic and **D** tumor tissues. **E** Difference in the immunoreactive score between paraneoplastic and tumor tissues. **F** Distribution of MT1A expression in PCa epithelial cells. **G** Volcano plot showing differentially expressed genes between high- and low-MT1A epithelial cells (MT1A is not shown in the plot due to the adjusted P-value of MT1A is too small). **H** Enrichment terms using the ClueGO. **I** Regulatory network of MT1A derived from the Bayesian network inference. **J** Correlations between MT1A and CSRP1 expression in pan-cancer. **K** Univariate Cox regression analysis of MT1A and CSRP1 in TCGA-PRAD, MSKCC2010, GSE70768, and DKFZ2018. **L** Correlations of MT1A with immune cells in PCa. Significance levels are denoted by the following symbols: *P < 0.05; **P < 0.01; ***P < 0.001

Both the classifier genes of ZHPS and the signature genes of ZHRRS contained MT1A, a metallothionein gene (Fig. 10A). Patients with advanced stages exhibited lower expression levels of MT1A (Fig. 10B). Decreased MT1A expression was observed in multiple tumor types (Supplementary Fig. 9). IHC analysis validated that the protein-level of MT1A was substantially lower in PCa tissues than in tumor-adjacent tissues (Fig. 10C, D, and E). Inflammatory pathways, such as interferon γ response, interferon α response, inflammatory response and complement pathways, were commonly upregulated in tumor samples with high MT1A expression (Supplementary Fig. 10). Both ComPPI and GeneMANIA revealed that the proteins interacting with MT1A included GPR50, GNAI1, ARRB1 and LAGE3 (Supplementary Fig. 11).

After processing the single-cell data (Supplementary Fig. 12), we noticed a considerable decrease of MT1A (Fig. 10F). Epithelial cells expressing MT1A and those not expressing MT1A were divided into two groups: high- and low-expression groups. A comparison between groups (high-expression vs low-expression groups) was carried out and 157 DEGs were identified (|Log2FC|> 0.25, adjusted *P* < 0.05) (Fig. 10G). The ClueGO tool revealed that these DEGs were mostly enriched in cellular zinc homeostasis and immune-regulatory pathways, such as positive regulation of T cell mediated immunity and regulation of dendritic cell differentiation (Fig. 10H). In order to further investigate the tumor-suppressing roles of MT1A, bayesian network models inferred that MT1A might positively regulate CSRP1 (Fig. 10I). Additionally, pan-cancer correlation analysis demonstrated that MT1A was most strongly correlated with CSRP1 in PRAD (Fig. 10J). Moreover, potential protective roles for MT1A and CSRP1 were observed in various cohorts (Fig. 10K). Positive correlations of MT1A expression with multiple immune cells, including the natural killer T, natural killer and activated CD8 T cells, were also prevalent (Fig. 10L).

## Discussion

Extensive molecular-genetic, biological and clinical heterogeneities are prominent hallmarks in PCa [50]. However, different clustering researches often yield distinct taxonomy results, due to differences in perspectives and scientific issues requiring clarification. For example, Meng et al. explored the immune subtypes of PCa by analyzing the activation status of the immune microenvironment [51]. They also constructed the prostate cancer multi-omics classification (PMOC) via multi-omics profile, which provided valuable insights for guiding precision treatment in PCa patients [52].

In the current study, we focused on zinc dyshomeostasis in PCa. Zinc reduction is prevalent in PCa patients, both in the glandular tissue and prostatic fluid [11]. Malignant epithelium typically exhibits lower high zinc levels than normal epithelium. One of the main causes of zinc depletion in prostate intraepithelial neoplasia (PIN) and PCa is the downregulation of ZIP1 expression [53]. Upregulated RREB1 expression during the early stages of PCa development leads to downregulation of ZIP1 expression and subsequent reduction in zinc ion concentration [54]. Different zinc regulators perform different functions in PCa. In the ZIP family, in addition to ZIP1 playing an oncogenic role, knockdown of ZIP4 significantly reduced the cell migration capacity of DU145 and 22Rv1, suggesting that ZIP4 may also play a role as an oncogene [19]. ZIP2 and ZIP3 function in the reabsorption and retention of zinc ions from prostate fluid, and significant downregulation of them in PCa was found in IHC assays [18]. Compared to the control group, the TRAMP mouse with ZNT7 knockout had a higher incidence of PIN at 6–8 weeks, as well as a higher incidence of PCa at 16 and 28 weeks. It is evident that ZNT7 could accelerate PCa progression [55]. It is thus apparent that zinc transporters and MTs are involved in tumor progression of PCa. Instead of focusing on individual genes, we investigated the impactful regulators of zinc homeostasis from a global perspective. Thus, a new molecular classification approach was created for a comprehensive exploration of zinc dyshomeostasis.

We observed different alterations of ZHRGs in diverse tumors, suggesting that the same zinc regulator may have distinct roles in different cancers. Take for example, MT1E can enhance the invasion and migration of glioma cells. In contrast, its decreased expression indicates an elevated likelihood of biochemical recurrence in PCa patients [56]. MT1H plays tumor-suppressing roles by regulating the Wnt/β-catenin signaling pathway in hepatocellular carcinoma [57], and interacts with EHMT1, which promotes its methyltransferase activity in prostate malignancies [58]. For lung cancer, MT1H overexpression results in enhanced cisplatin resistance [59]. In the aspect of genomic mutations, AP3B1 has the highest mutation frequency across all tumors, especially in UCEC. We also found that SNVs may not be a crucial cause of ZHRG alterations in PCa, owning to their low mutation rate. The lower expression of genes in the metallothionein family can be partially explained by elevated promoter methylation levels. The complicated roles of ZHRGs, which vary according to different tumor environments, warrant further research.

In the present study, two distinctly different subtypes were identified based on the expression of ZHRGs, and it was validated by the NTP algorithm that the clustering was robust in different cohorts and platforms. ZHPS2 exhibited greater malignancy in terms of several clinicopathological features, which aligns with its inferior prognosis. As analyzed earlier, ZHPS2 was characterized by a dramatic mutational landscape. Through functional exploration analysis, we could conclude that ZHPS2 was in the state of zinc dyshomeostasis, while ZHPS1 maintained zinc homeostasis. Notably, compared with ZHPS1, ZHPS2 had higher expression of immune checkpoints, suggesting impaired immune functions. In the innate immunity, dysregulation of zinc homeostasis, such as zinc deficiency, inhibits macrophage phagocytosis and polymorphonuclear leukocyte chemotaxis [60]. Furthermore, zinc deficiency diminishes the pro-inflammatory cytokine production in monocytes [61]. Zinc homeostasis also plays an essential role in the adaptive immune system, involving TCR-, IL-1R-, IL-2R- and IL-6R-mediated T cell signaling pathways [62]. Altogether, the dysregulation of zinc homeostasis disrupts the immune function and creates an immunosuppressive microenvironment.

Notably, there were elevated TMB in ZHPS2, along with the increased MSI. Patients with high TMB or MSI tend to be highly sensitive to immunotherapy [63], which suggests that ZHPS2 is an appropriate candidate for immunotherapies. Also, docetaxel was speculated to be more effective in ZHPS2. The application of docetaxel can enhance the antitumor immune response [64]. Therefore, a combination strategy of docetaxel and immune-based therapies may bring the zinc dyshomeostasis group, ZHPS2, more clinical benefits. High response rates to olaparib have been observed in patients with mutations in BRCA1, BRCA2, ATM, PALB2, FANCA, and CHEK2 [65]. The higher proportion of BRCA2 and ATM aberrations in ZHPS2 accounted for its greater sensitivity to olaparib. Nevertheless, further investigation is needed to determine whether and how zinc dyshomeostasis leads to changes in the response to docetaxel and olaparib.

After using the WGCNA algorithm to identify the hub genes strongly associated with ZHPS2, univariate Cox regression analysis then filtered out the genes unrelated to prognosis. We entered the remaining genes into a pipeline consisting of 10 machine learning algorithms for comparison, and ultimately obtained the optimal model using the elastic network (α = 0.1). The resulting ZHRRS not only possessed a stable performance in different PCa cohorts, but was also non-inferior to multiple previously published signatures. Astonishingly, ZHRRS can even be extended to the prognostic prediction of most other tumors. The nomogram based on the signature and several clinicopathological features also performed well in prognosis assessment. We therefore summarized that the quantitative instrument, ZHRRS, has further potential for clinical practice.

Subsequently, MT1A emerged as a critical gene due to its appearance in both the ZHPS classifier and ZHRRS model. There was a significant reduction of MT1A expression levels in the advanced PCa, and we also confirmed the decrease of MT1A at the protein-level. From the exploration of single-cell and bulk sequencing data for MT1A, the potential tumor-suppressor and pro-inflammatory roles were discovered. Similar downward trends in expression have been observed in other tumors. In lung cancer, low expression of MT1A is associated with the tumorigenesis [66]. The high methylation level of 5′ CpG island is the leading cause of MT1A aberrant silencing [67]. Similarly, significant decreases in MT1A expression have been observed in oral squamous cell and papillary thyroid carcinomas [68, 69]. However, the functions of MT1A in malignant transformation and development have been less studied. CSRP1, a cysteine-rich protein that is inferred as a positive regulator downstream of MT1A according to the BN, has been implicated in influencing the progression of PCa [70]. In addition, we observed upregulation of ECRG4, FRZB, and CYTL1 in MT1A-high-expressing cells. As a tumor suppressor, ECRG4 is downregulated in cancers due to promoter methylation [71, 72], and its immune activation and tumor inhibition functions may be attributed to its TLR4-targeted internalization domain [73]. Furthermore, FRZB considerably suppresses tumor growth and invasion through the inhibition of Wnt/β-catenin pathway [74]. The antitumor activities of CYTL1 involve the reversal of glycometabolism reprogramming [75]. MT1A may also alter the TME, making it highly susceptible to a pro-inflammatory state, which is another mechanism of tumor remission.

The following deficiencies are still existing in our study. On the one hand, the publicly available datasets used in our study were retrospectively designed. In order to validate the ZHPS classifier and ZHRRS model, conducting a prospective study using an in-house cohort is necessary. On the other hand, further evidence to support the biological roles of MT1A in PRAD is required through in vivo and in vitro experiments.

## Conclusions

From a comprehensive perspective on zinc homeostasis, we identified a zinc dyshomeostasis cluster, named ZHPS2. This subtype had a significantly worse prognosis, while it could benefit more from docetaxel and olaparib treatments. Benefiting from multifariously machine learning approaches, the ZHRRS model was thus generated. It is not hard to see the powerful signature is promising for clinical translation and application. Ultimately, the potential mechanisms regarding MT1A have been dissected. Collectively, our findings provide novel insights into the role of zinc homeostasis in PCa, which will translate into favorable clinical practices in the future.

### Supplementary Information

Supplementary material 1.

Supplementary material 2.

## Data Availability

The datasets involved in this study are available in TCGA (http://portal.gdc.cancer.gov/), GEO (https://www.ncbi.nlm.nih.gov/geo/), cBioPortal (https://www.cbioportal.org/), and UCSC Xena (https://xena.ucsc.edu/).
