# Supplementary material for: Deciphering the role of zinc homeostasis in the tumor microenvironment and prognosis of prostate cancer
Source: Discov Oncol. 2024 Jun 4;15:207. doi: 10.1007/s12672-024-01006-z (PMC11150232; doi:10.1007/s12672-024-01006-z)
Supplement: Supplementary file 1 — Supplementary material 1. [file 12672_2024_1006_MOESM1_ESM.docx]

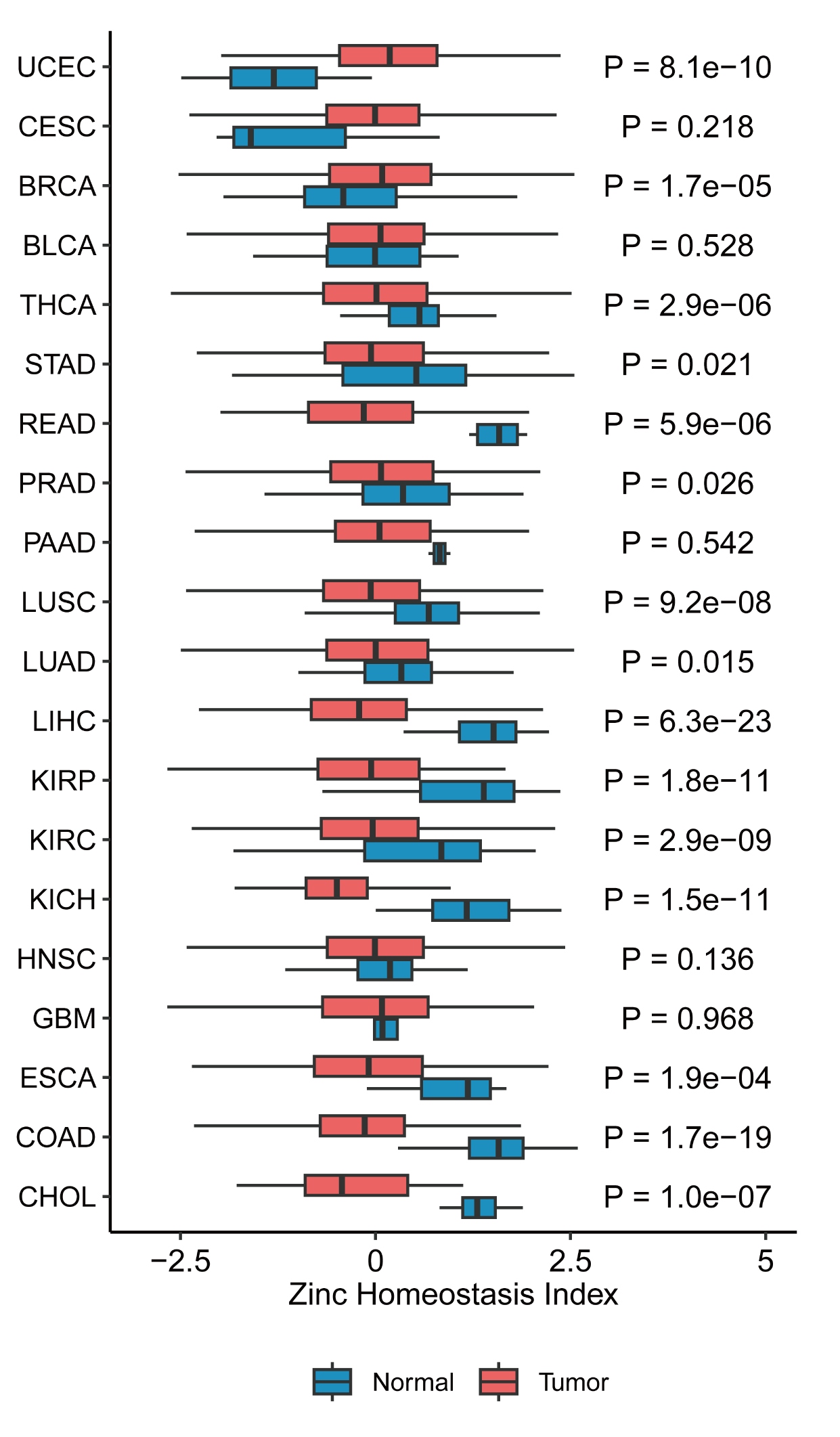


**Fig. S1** The comparison of zinc homeostasis index (tumor versus normal adjacent samples).


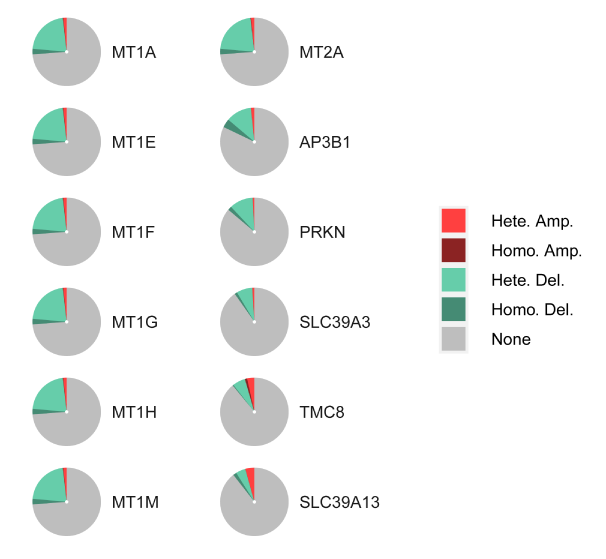


**Fig. S2** ZHRG CNV amplifications and deletions in PRAD**.**

**
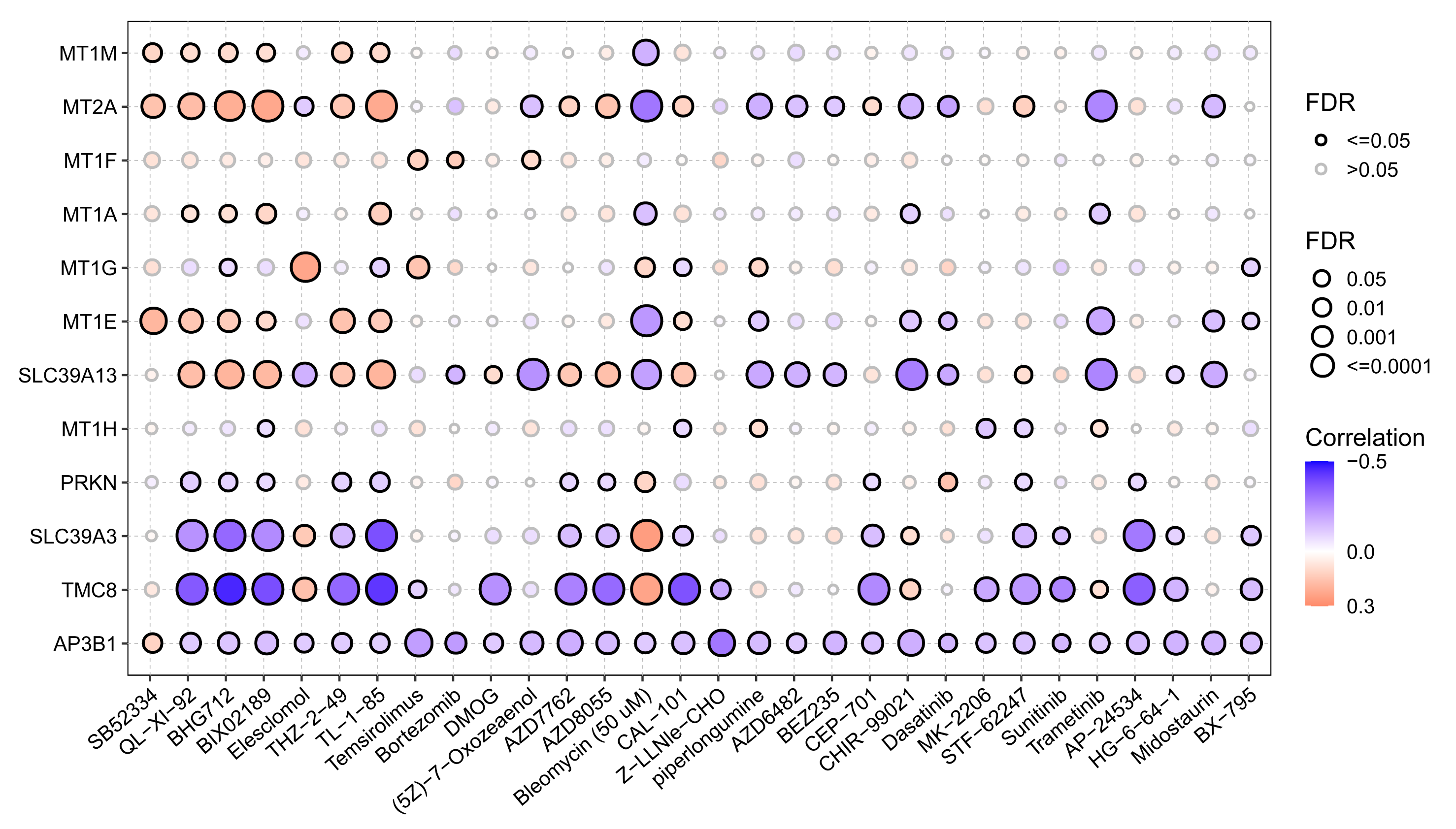
**

**Fig. S3** Correlations between SLC39A13 expression levels and the IC50 of drugs based on the GDSC database.


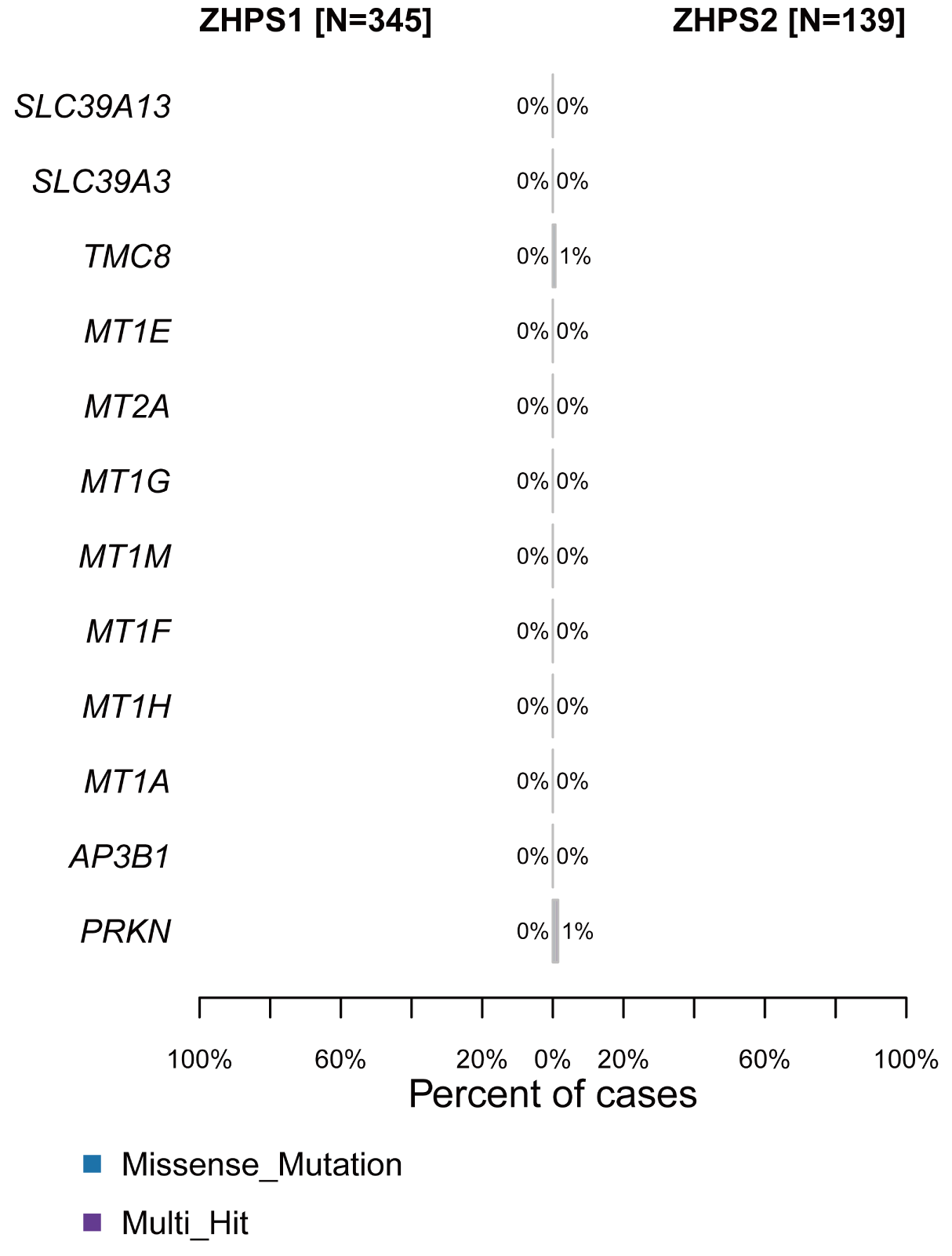


**Fig. S4** SNV landscapes of ZHRGs in ZHPS1 and ZHPS2.


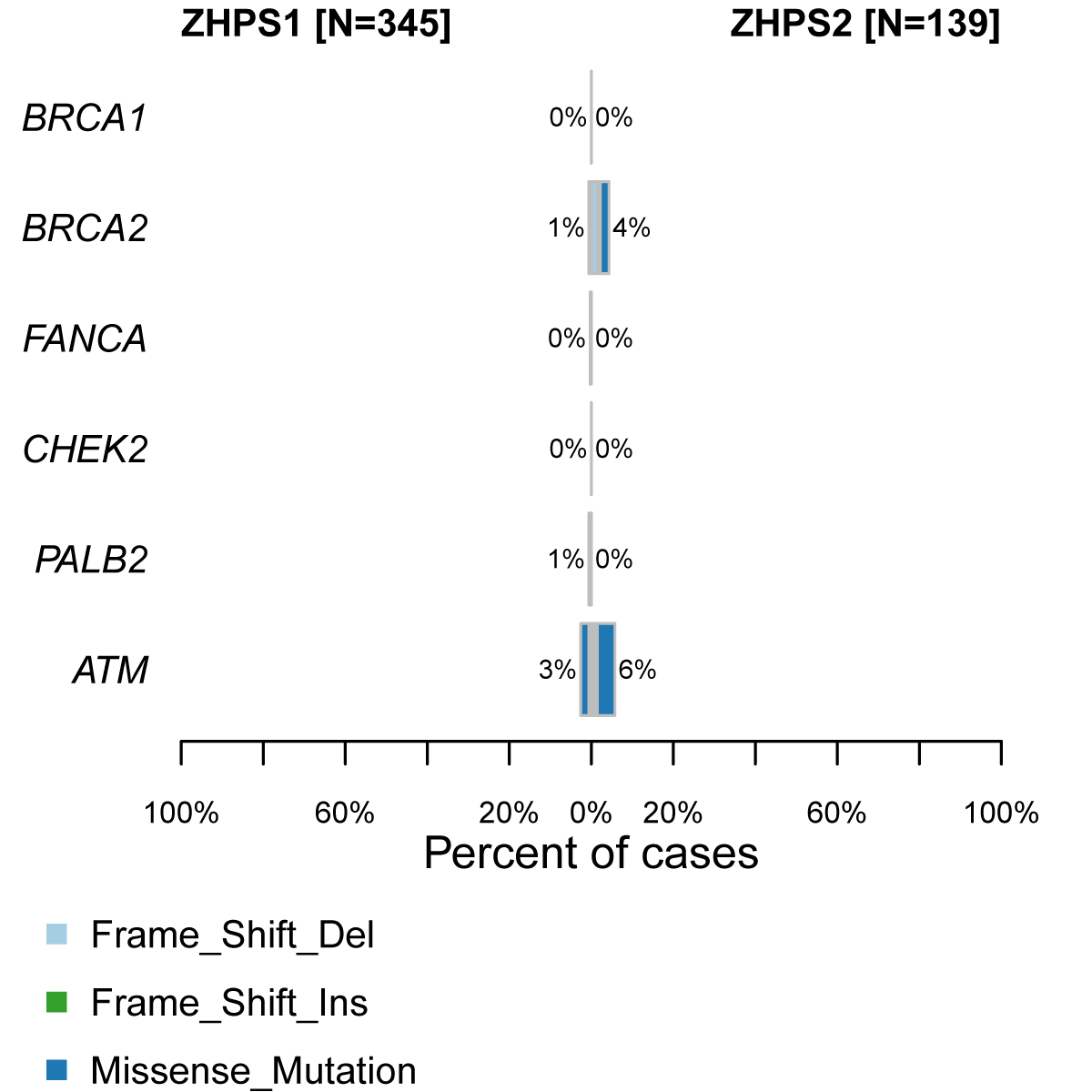


**Fig. S5** SNV landscapes of DNA repair-related genes in ZHPS1 and ZHPS2.


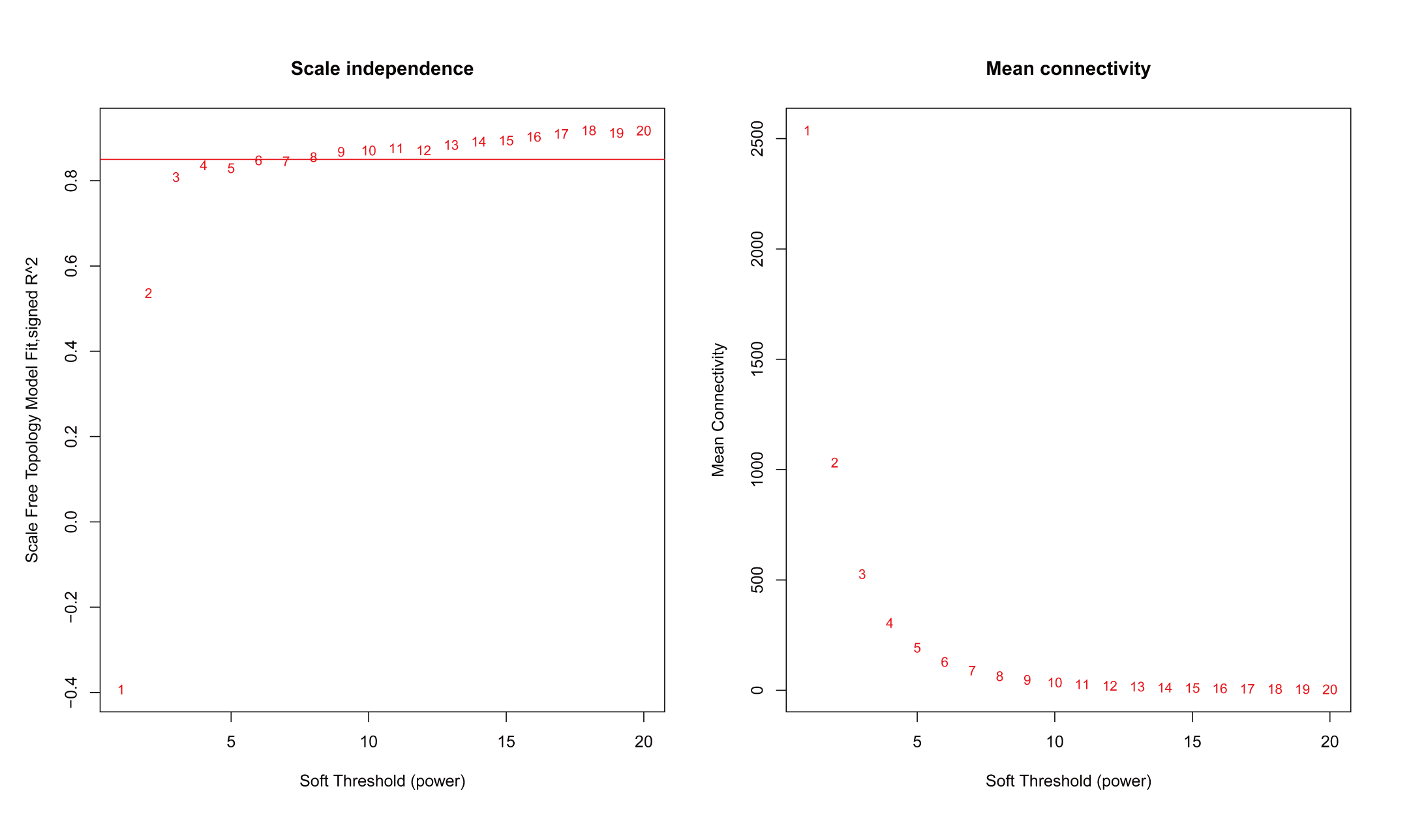


**Fig. S6** The scale-free topology model fit (R^2^) and mean connectivity in different soft-threshold powers.


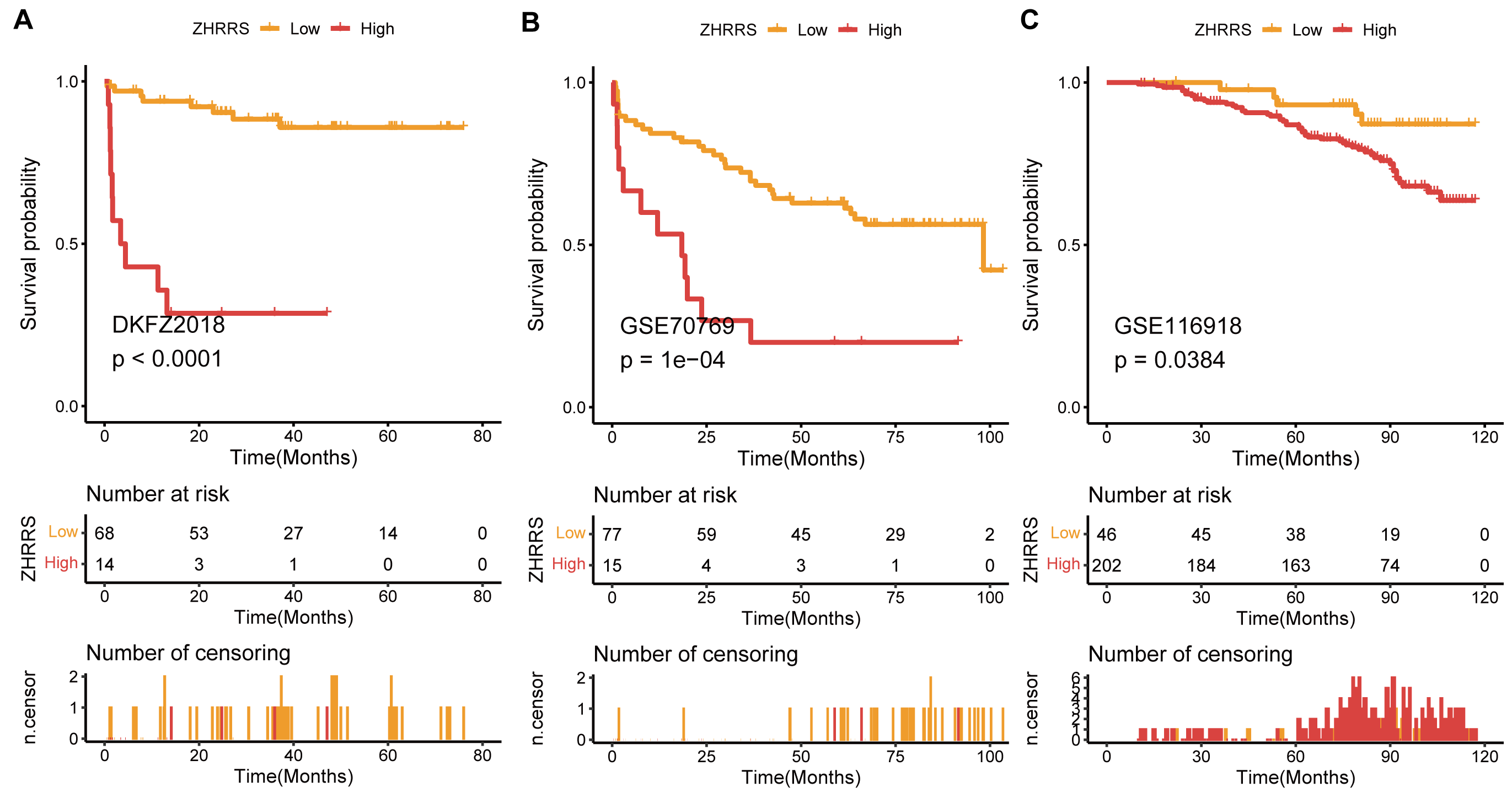


**Fig. S7** Kaplan-Meier curves of BCR in DKFZ2018, GSE70769, and GSE116918.


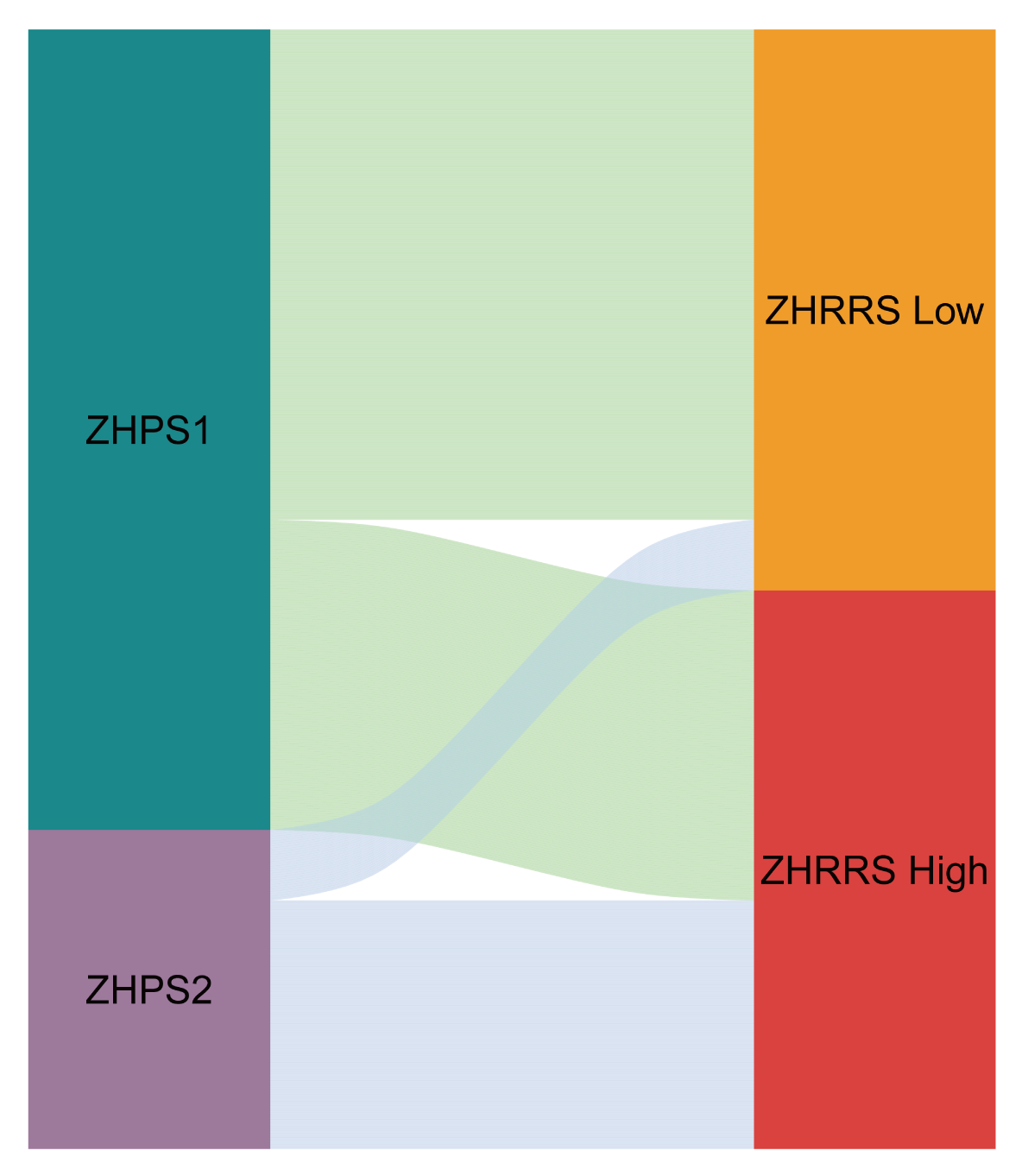


**Fig. S8** Sankey diagram showing the percentages of patients from ZHPS toward ZHRRS.


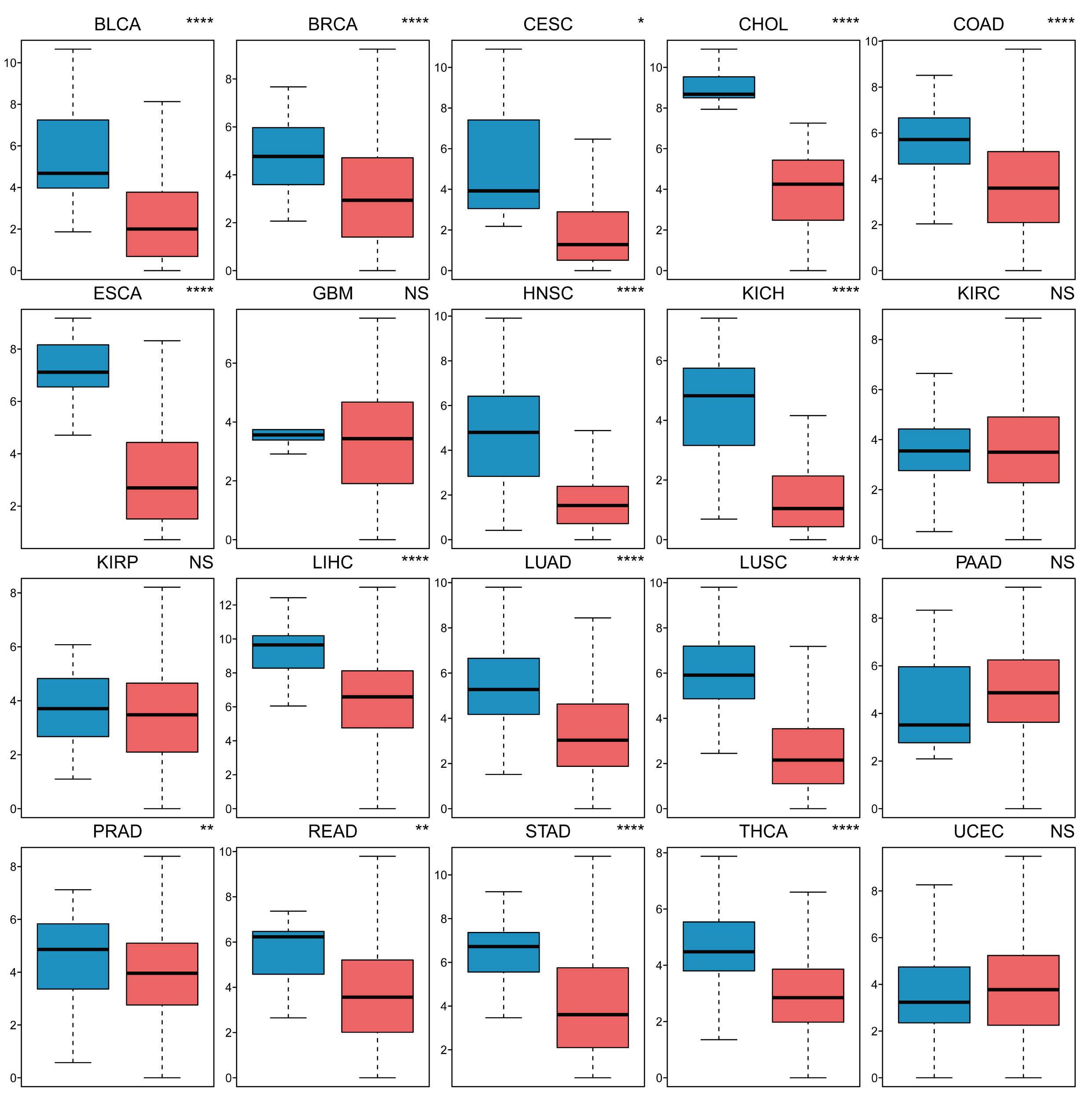


**Fig. S9** MT1A expression comparisons between cancerous and paracancerous tissues. P-value symbols are indicated: **P* < 0.05; ***P* < 0.01; ****P* < 0.001; *****P* < 0.0001.


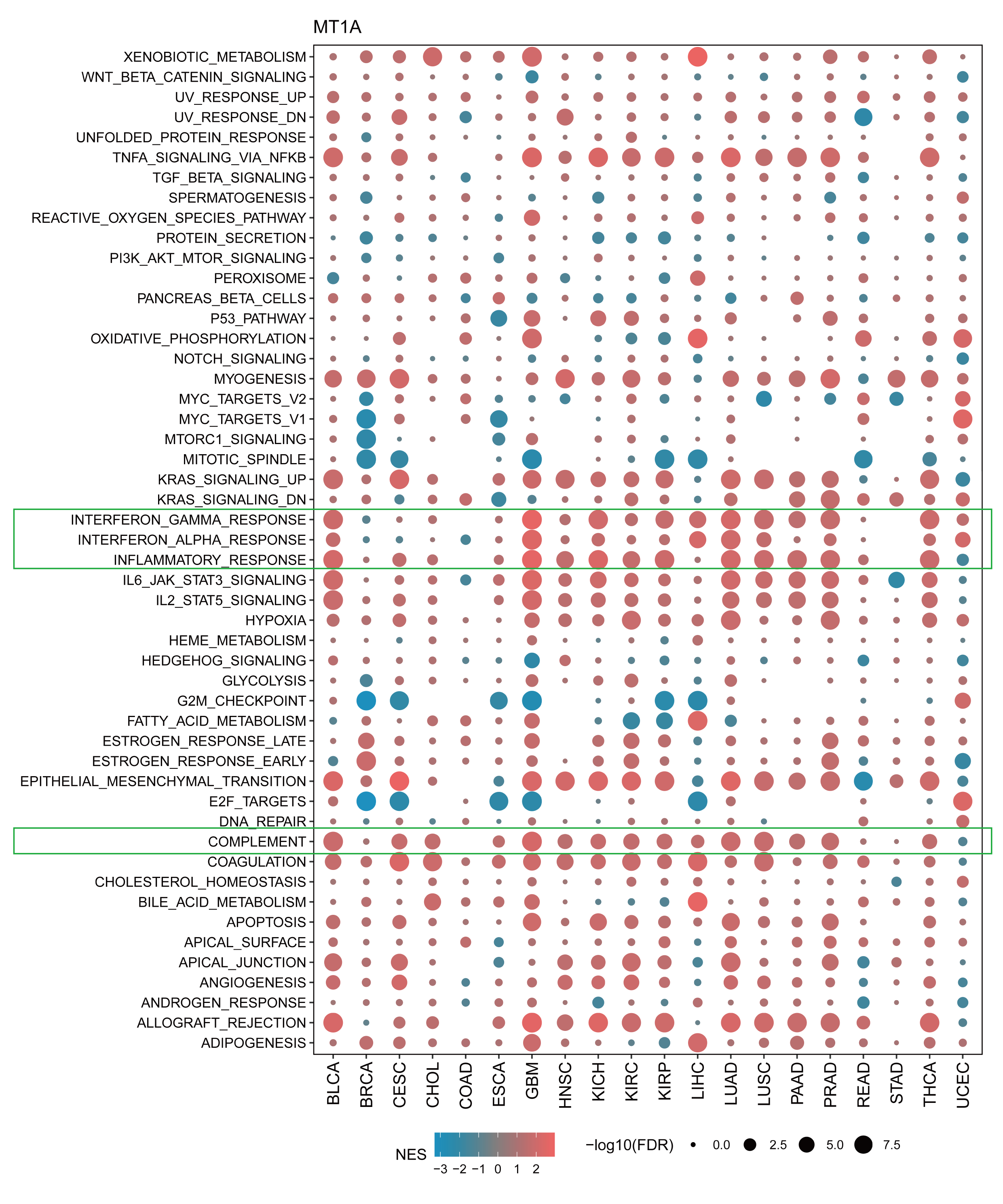


**Fig. S10** Hallmark pathway differences between high-MT1A and low-MT1A patients (NES: normalized enrichment score).


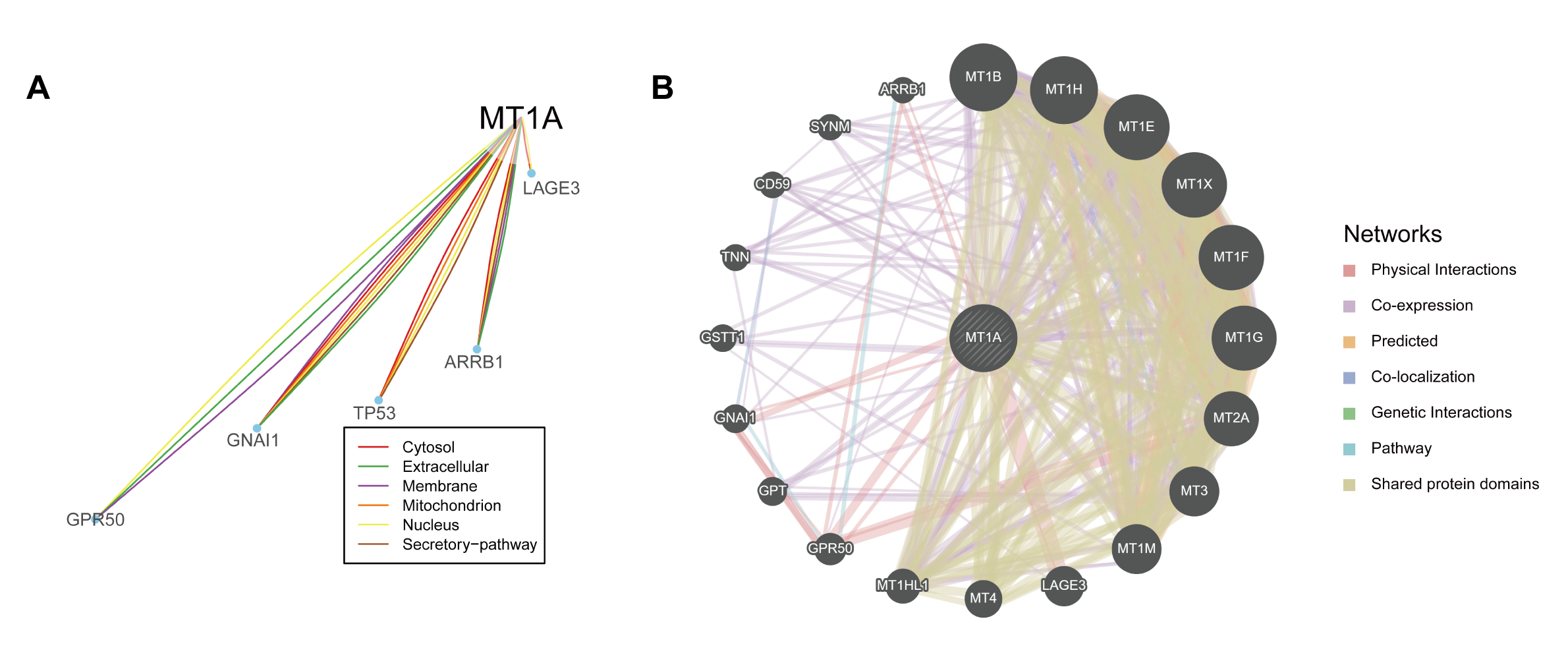


**Fig. S11** PPI networks with MT1A (left: ComPPI database; right: GeneMANIA).


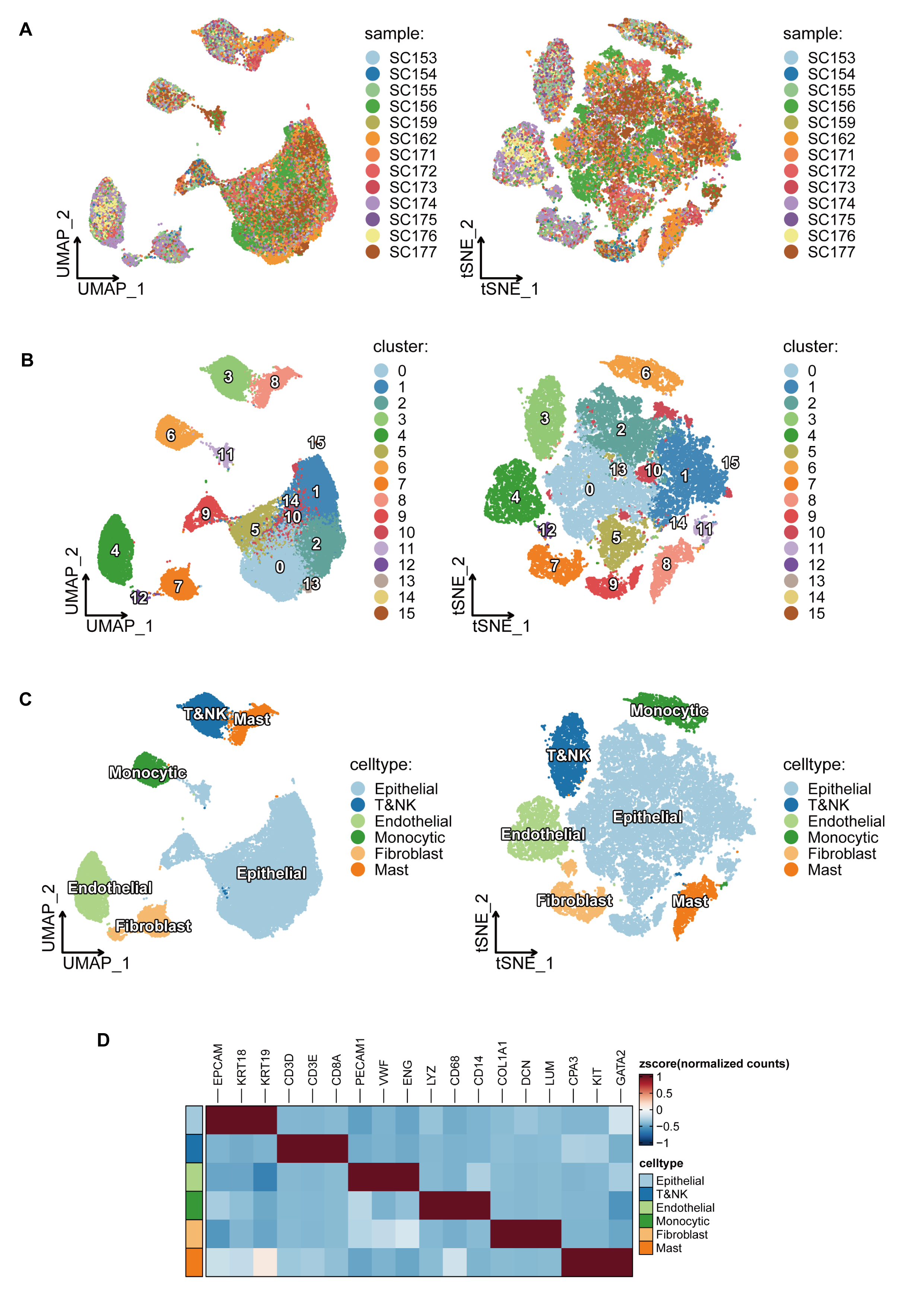


**Fig. S12** Processing of single-cell data. (**A**) UMAP and tSNE plots grouped by different samples. (**B**) UMAP and tSNE plots grouped by different clusters. (**C**) UMAP and tSNE plots grouped by different celltypes. (**D**) Classical marker gene expression in each celltype.
